# Spatial covariance of mGluR5 density and structural degeneration in behavioral variant frontotemporal degeneration

**DOI:** 10.1162/IMAG.a.1358

**Published:** 2026-09-10

**Authors:** Melanie A. Matyi, Maggie K. Pecsok, Christopher A. Olm, Hamsanandini Radhakrishnan, Laynie Dratch, Vivianna M. Van Deerlin, Emma Rhodes, Jeffrey S. Phillips, Philip A. Cook, James C. Gee, David J. Irwin, Corey T. McMillan, David Roalf, Lauren Massimo

**Affiliations:** Penn Frontotemporal Degeneration Center, Department of Neurology, School of Medicine, University of Pennsylvania, Philadelphia, PA, United States; Brain Behavior Lab, Neurodevelopmental and Psychosis Section, Department of Psychiatry, School of Medicine, University of Pennsylvania, Philadelphia, PA, United States; Department of Pathology and Laboratory Medicine, University of Pennsylvania, Philadelphia, PA, United States; Penn Image Computing and Science Laboratory, Department of Radiology, University of Pennsylvania, Philadelphia, PA, United States; Department of Biobehavioral Health Sciences, School of Nursing, University of Pennsylvania, Philadelphia, PA, United States

**Keywords:** behavioral variant frontotemporal degeneration, glutamate, spatial covariance, executive function

## Abstract

Post-mortem human brain tissue and *in vivo* imaging studies of behavioral variant frontotemporal degeneration (bvFTD) suggest that metabotropic glutamate receptors (mGluR) may be vulnerable to the disease process. However, these studies lack evidence demonstrating how loss of mGluR relates to clinical features of bvFTD, hindering efforts to identify appropriate therapeutic strategies. Thus, we leveraged existing normative receptor density maps, and a large cohort of individuals with bvFTD to elucidate relationships among structural degeneration, glutamatergic receptor density, and clinical features. We obtained receptor density maps from the neuromaps repository, which contains positron emission tomography-derived receptor maps across many neurotransmitter systems, including mGluR5 and N-methyl-D-aspartate (NMDA). We quantified the spatial covariance of structural degeneration and glutamate receptor density maps in 106 individuals with bvFTD and 165 cognitively unimpaired (CU) individuals. Covariance of each participant’s regional cortical volumes and normative receptor density in each region of the Schaefer 100 (7 network) parcellation was computed. Negative covariance indicates a high spatial correspondence such that regions with greater structural degeneration also have high receptor density. Within bvFTD, we tested whether greater spatial covariance of structural degeneration and receptor density maps were associated with disease stage and severity, executive function, and neuropsychiatric symptoms. Comparing bvFTD with CU, we observed significant spatial covariance of structural degeneration and mGluR5, but not NMDA. In bvFTD, greater covariance of structural degeneration and mGluR5 maps was associated with more advanced disease stage (*p* = .003) and severity (*p* < .001), and worse category (*p* < .001) and letter-guided (*p* < .001) fluency and digit span backward (*p* = .0257) but not neuropsychiatric symptom severity. Neurodegeneration in bvFTD appears to preferentially affect cortical regions with higher normative mGluR5 density, as reflected by spatial covariance between structural degeneration and mGluR5 maps in a clinically well-characterized cross-sectional cohort. Furthermore, greater covariance of mGluR5 density and structural degeneration with both disease severity and executive dysfunction elucidates potential neural mechanisms of core clinical features of bvFTD. Future work examining *in vivo* glutamate could provide further insight into the natural history of glutamate dysfunction in bvFTD, facilitating testing of glutamatergic therapies.

## 1 Introduction

Converging evidence indicates reduced glutamatergic functioning in behavioral variant frontotemporal degeneration (bvFTD): lower glutamate levels and fewer glutamatergic neurons and receptors are observed in bvFTD compared with cognitively unimpaired (CU) individuals (Huber et al., 2022; Murley & Rowe, 2018). Postmortem studies demonstrate loss of glutamatergic neurons in the frontal and temporal cortices and thalamus (Ferrer, 1999), fewer alpha-amino-3-hydroxy-5-methyl-4-isoxazolepropionic acid (AMPA) receptors, and inconsistent evidence of N-methyl-D-aspartate (NMDA) receptors changes in the frontal and temporal cortices (Bowen et al., 2008; Procter et al., 1999). Other regions affected by bvFTD pathology, including the anterior cingulate and insular cortices, also show pronounced glutamatergic deficits, with significantly greater loss of glutamate, relative to non-glutamate neurons in bvFTD as compared with CU (53% loss) and Alzheimer’s disease (41% loss) (Dijkstra et al., 2018; Santillo et al., 2013). Together, post-mortem findings provide strong evidence for glutamatergic system dysfunction in bvFTD; however *in vivo* studies remain limited, and the clinical consequences of these deficits are largely unknown.

*In vivo* proton magnetic resonance spectroscopy (^1^HMRS) studies report lower glutamate levels in bvFTD than in CU in the mid-frontal lobe (Ernst et al., 1997) and the inferior frontal gyrus (Murley et al., 2020, 2022). Lower neurometabolite levels, particularly glutamate levels, strongly correlate with clinical features such as poor verbal fluency and reduced motivation (Murley et al., 2022). Beyond lower glutamate levels, an *in vivo* positron emission tomography (PET) study showed markedly lower mGluR5 availability in bvFTD than in CU (Leuzy et al., 2016). Despite a small sample (5 bvFTD, 10 CU), mGluR5 availability was approximately 65% (4 standard deviations) lower in bvFTD and spatially overlapped with gray matter atrophy (Leuzy et al., 2016). Collectively, these findings indicate clear but under-characterized glutamatergic system deficits in bvFTD.

The glutamatergic system is highly complex, with glutamate acting on fast-acting ionotropic (e.g., NMDA, AMPA, Kainate) and slower acting metabotropic (mGluR) receptors, with mGluRs being further classified into type I (excitatory), type II (inhibitory), and type III (primarily inhibitory). Although reduced mGluR5 availability has been demonstrated *in vivo* in bvFTD (Leuzy et al., 2016), similar examination of NMDA has not yet been undertaken in bvFTD, or frontotemporal lobar degeneration (FTLD) more broadly. *In vivo* measurement of the glutamatergic system is further constrained by methodological limitations. PET is restricted to a single tracer, and thus, one receptor, per measurement, while ^1^HMRS measures the levels of many neurometabolites, including glutamate, but is spatially limited to selected brain regions, constraining prior findings to a limited set of regions being examined (Ernst et al., 1997; Murley et al., 2020). As a result, existing approaches provide a limited view of glutamatergic dysfunction in bvFTD.

Recent publicly available tools and curated brain map datasets, such as neuromaps (Markello et al., 2022), enable anatomical alignment of normative receptors maps to standard atlas space and quantitative comparison of brain maps across modalities and cohorts. Neuromaps have been used to integrate PET-based glutamate receptor maps with molecular imaging of glutamate (Pecsok et al., 2025), link brain-related diseases to neurotransmitter receptor systems (Hansen et al., 2022), and identify biomarkers of disease risk and treatment responsiveness (Tarchi et al., 2025). A key advantage of neuromaps is that existing unimodal datasets can be enriched with a broad array of normative brain maps. Here, we leveraged glutamate receptor maps (Hansen et al., 2022) of mGluR5 (DuBois et al., 2016; Smart et al., 2019) and NMDA (Galovic et al., 2021, 2023; McGinnity et al., 2014) together with our large, well-characterized cohort of individuals with bvFTD, to test whether the normative spatial distribution of glutamatergic receptors overlaps with patterns of structural degeneration. Substantial overlap between receptor density and structural degeneration suggests that structural degeneration is either a consequence of, or contributor to, glutamatergic dysfunction, providing mechanistic insight into bvFTD.

Based on prior evidence of reduced mGluR5 availability in bvFTD relative to CU, we hypothesized that individuals with bvFTD would show greater correspondence between mGluR5 density and structural degeneration, compared with CU. We further hypothesized that, within bvFTD, greater covariance of mGluR5 and structural degeneration would be associated with more advanced disease stage and severity, executive dysfunction, and neuropsychiatric symptoms but not visuospatial functioning. To demonstrate specificity of findings to mGluR5, we evaluated additional receptor maps. NMDA was evaluated as the only other glutamate-related map. Given inconsistent evidence, we did not make any specific hypotheses about NMDA associations with clinical outcomes. Vesicular acetylcholine transport (VAChT) was evaluated to assess specificity, as VAChT is a component of the acetylcholine neurotransmitter system that is not highly impacted in bvFTD (Murley & Rowe, 2018), and thus, we expected no clinical associations. Finally, to test the robustness of mGluR5-related findings, we examined serotonin receptor 6 (5HT6), a non-glutamatergic receptor with the most similar spatial distribution to mGluR5 among receptor maps available in neuromaps. We restricted analyses to this subset of hypothesis-driven maps, rather than an omnibus evaluation of all available maps to reduce the risk of type I errors due the highly collinear spatial properties of neural maps, particularly neurotransmitter maps (Hansen & Misic, 2025).

## 2 Methods

### 2.1 Participants

The Penn Integrated Neurodegenerative Disease Database (INDD) (Toledo et al., 2014) was queried for individuals with a clinical diagnosis of probable or definite bvFTD, T1-weighted (T1w) MRI acquired using one of the protocols described below, and complete clinical data within 9 months of MRI acquisition. A total of 110 individuals met these criteria. Four individuals were suspected to have a disease other than bvFTD that may be driving symptoms, including amyotrophic lateral sclerosis (ALS), dementia with Lewy bodies (DLB), frontal variant AD, and progressive supranuclear palsy (PSP). To focus on bvFTD, these four individuals were excluded from analyses. All bvFTD participants were evaluated at the University of Pennsylvania Frontotemporal Degeneration Center by experienced clinicians using published consensus criteria (Rascovsky et al., 2011). Participants and their legal representatives participated in an informed consent procedure approved by the institutional review board at the University of Pennsylvania. For participants with multiple eligible visits, the earliest was analyzed. A comparison group of 165 age and MRI protocol-matched CU with a normal cognitive examination and no history of neurologic or psychiatric conditions was also included. Propensity matching was performed by calculating the distance between participants via generalized linear model with a probit link. To be matched, participants must have a standardized distance less than 0.2 on age and an exact match on MRI protocol (Austin, 2011). Each member of the bvFTD group was matched to one or more CU participants.

We report the proportion of bvFTD participants with confirmed neuropathology (n = 40, 38% of sample) and known pathogenic genetic variants associated with FTLD (29% of sample) in Table 1. Given the small proportion of our sample with confirmed neuropathology and/or pathogenic genetic variants, we were not powered to examine associations stratified by genetic status or by proteinopathy. Individuals with bvFTD underwent genetic evaluation for *C9orf72* hexanucleotide repeat expansion and pathogenic variants in *MAPT*, *GRN*, *TARDBP*, and *VCP*. Genomic DNA was extracted from peripheral blood or frozen brain tissue samples (Caswell et al., 2019). The presence of a *C9orf72* repeat expansion was evaluated using a modified repeat-primed polymerase chain reaction (Suh et al., 2015). *GRN, MAPT, TARDBP*, and *VCP* variants were identified from whole genome sequencing, whole exome sequencing, or targeted neurodegeneration sequencing panel datasets (Toledo et al., 2014). The bvFTD cases that did not have positive tests for *C9orf72* repeat expansions or pathogenic genetic variants were defined as apparently sporadic bvFTD. A subset of individuals with bvFTD had a neuropathological diagnostic evaluation for FTLD (Dickson et al., 2011; Mackenzie et al., 2011) available. FTLD-TAU pathology included Pick disease, argyrophilic grain disease, progressive supranuclear palsy, tauopathy unclassifiable, corticobasal degeneration, and globular glial tauopathy (Dickson et al., 2011). FTLD-TDP pathology included types A–E (Lee et al., 2017; Mackenzie et al., 2011). Autopsies were performed using standard protocols (Lee, 2018; Toledo et al., 2014) at the Center for Neurodegenerative Disease Research, University of Pennsylvania.

**Table 1.** Demographic and clinical characteristics of the study sample.

Characteristic	bvFTDN = 106^a^	CUN = 165^a^	*p*-value^b^
Sex (female)	39 (37%)	102 (62%)	<.001
Age (years)	64.14 (7.21)	62.57 (6.78)	.049
Education (years)	15.75 (2.57)	15.60 (2.75)	.7
Acquisition protocol (1)	38 (36%)	139 (84%)	<.001
CDR global			<.001
0	0 (0%)	165 (100%)	
0.5	28 (26.4%)	0 (0%)	
1	60 (56.6%)	0 (0%)	
2	16 (15.1%)	0 (0%)	
3	2 (1.9%)	0 (0%)	
Pathogenic genetic variant
Apparently sporadic	75 (70.8%)	—	
*C9orf72*	21 (19.8%)	—	
*MAPT*	4 (3.8%)	—	
*GRN*	4 (3.8%)	—	
*TARDBP*	1 (0.9%)	—	
*VCP*	1 (0.9%)		
Genetic or autopsy-confirmed pathology
TAU	9 (8.5%)	—	
TDP	31 (29.2%)	—	
Unknown	66 (62.3%)	—	
Clinical measure			
FTLD CDR sum of boxes	8.00 (6.50, 10.50)	—	
Digit span backward correct	4.63 (2.64)	—	
Category-guided fluency	8.99 (5.23)	—	
Letter-guided fluency	8.48 (5.79)	—	
Neuropsychiatric inventory severity	9.00 (5.00, 15.00)	—	
Figure copy	14.17 (3.69)	—	

_a_ n (%); mean (SD); median (Q1, Q3).

_b_ Pearson’s chi-squared test; Wilcoxon rank sum test; Fisher’s exact test.

### 2.2 MRI acquisition

Participants underwent MRI an average of 1 month after neuropsychological testing (median = 0, same day; range = 0–8 months). T1w MPRAGE images were acquired using one of two comparable protocols. Protocol 1: 3T Siemens TIM Trio scanner, 8 channel head coil, axial plane with repetition time = 1620 ms, echo time = 3.87 ms, slice thickness = 1.0 mm, flip angle = 15 degrees, matrix = 192 × 256, in-plane resolution = 0.977 × 0.977 mm. Protocol 2: 3T Siemens Prisma Fit scanner, 64 channel head coil, sagittal plane with repetition time = 2400 ms, echo time = 1.96 ms, slice thickness = 0.8 mm, flip angle = 8 degrees, matrix = 320 × 320, in-plane resolution = 0.8 × 0.8 mm. Metrics derived from T1w images were adjusted for protocol.

### 2.3 MRI data processing

Regional brain volumes were derived from T1w images processed using the Advanced Normalization Tools (ANTs) antsCorticalThickness.sh pipeline (Tustison et al., 2014) (https://github.com/ftdc-picsl/antsct-aging [v0.3.3-p01]). Briefly, intensity nonuniformity was corrected with N4 bias correction (Tustison et al., 2010). Atropos segmentation used template-based priors to segment the brain into six classes (cortical gray matter (GM), subcortical GM, white matter (WM), cerebrospinal fluid (CSF), brainstem, and cerebellum) (Avants et al., 2011). Then, a nonlinear registration aligned the bias-corrected brain image to the group template (Tustison et al., 2019). Regions of interest (ROI) were then transformed from group template space to native T1w space using the inverse of the native T1w space template warp. Template-based ROIs are large relative to brain cortex and so, once in native T1w space, ROIs were masked by the individual’s cortex. Cortex was defined as voxels with a cortical thickness value >0.0001 mm, estimated using DiReCT (Das et al., 2009), to remove spurious voxels. Volumes of ROIs were calculated and used in all analyses.

Primary analyses used the Schaefer 100 (7 network) cortical parcellation (Schaefer et al., 2018; Yeo et al., 2011). Supplementary analysis examined higher resolution Schaefer parcellations (200 and 400) and an anatomically based atlas, the Lausanne atlas (Cammoun et al., 2012), a 219 region parcellation that provides intermediate spatial resolution.

### 2.4 Covariance calculation

Structural degeneration was quantified using w-scores, adjusting for T1w protocol, age at MRI, and intracranial volume (ICV) (Burke et al., 2022; Joie et al., 2012). For each region, linear regression models were fit to CU participants, with regional volume predicted by protocol, age, and ICV. These models were then applied to the full sample. W-scores were computed as the difference between observed and fitted values. Lower w-scores indicate lower than expected regional volume given protocol, age, and ICV.

Receptor density maps were obtained from neuromaps (DuBois et al., 2016; Galovic et al., 2023; Hansen et al., 2022; Markello et al., 2022; Smart et al., 2019). Publicly available parcellated maps of mGluR5, NMDA, VAChT, and 5HT6 maps (Hansen et al., 2022) corresponding to Schaefer 100, 200, and 400 parcellations and the Lausanne 125 (219 regions) atlas were used in analyses. A table detailing the ligands used, sample sizes, and demographic characteristics for each map obtained from neuromaps is included in the Supplementary Table S1.

Covariance of receptor maps and structural degeneration were quantified by computing, for each participant, correlation between their regional w-scored structural degeneration map, and each receptor density map, consistent with prior work (Hansen et al., 2022; Luo et al., 2024; Pecsok et al., 2025). Specifically, a vector of w-scored regional volumes (1 x number of regions) was correlated with a vector of regional receptor densities (1 x number of regions), resulting in one correlation coefficient for each receptor (e.g., mGluR5, NMDA). Negative correlations indicate that regions with a higher receptor density exhibit greater structural degeneration (i.e., co-localization of structural degeneration and receptor-rich regions), whereas positive correlations indicate relative sparing of regions with high density of receptors. Correlations near zero reflect little spatial correspondence between structural degeneration and receptor distribution. This approach preserves spatial correlation values at the individual level enabling assessment of how the correspondence between structural degeneration and receptor distribution relates to clinical features. To test group-level differences in map covariance by clinical phenotype and disease stage, we also generated group-averaged structural degeneration maps for comparison with receptor maps.

### 2.5 Clinical assessments

Executive function was evaluated with verbal fluency measures and digit span backward (Aita et al., 2019; Lezak et al., 2012). Category-guided fluency required participants to generate as many unique words as possible for animals and vegetables within 60 seconds per category. Letter-guided fluency required participants to generate as many unique words as possible, excluding proper nouns and numbers, starting with the letter “F” within 60 seconds. Scores for both tests were the total number of correct, non-repeated responses. Digit span backward (DSB) assessed working memory by requiring participants to repeat increasingly longer sequences of digits in reverse order. The score was the number of correct trials completed before two consecutive errors.

Neuropsychiatric symptoms were indexed by the total severity score from the Neuropsychiatric Inventory (NPI) (Cummings, 1997). Visuospatial functioning was assessed with the Benson Complex Figure Copy test (Possin et al., 2011), in which participants copy a complex figure and receive up to 2 points (accuracy and placement) for each of 8 figure elements, yielding a maximum score of 16.

Disease stage was indexed using the Clinical Dementia Rating (CDR) Global score, and disease severity using the CDR plus FTLD sum of boxes (SOB) score. The CDR Global score reflects overall dementia stage, including clinician ratings of participants’ functioning across six domains: memory, orientation, judgment and problem-solving, community affairs, home and hobbies, and personal care. The CDR plus FTLD adds two domains relevant to FTLD: behavior (including comportment and personality) and language. The FTLD CDR SOB score can range from 0 to 24. Only two individuals with bvFTD had a CDR Global score of 3 (see Table 1), and thus we combined CDR Global scores of 2 and 3 resulting in three subgroups of individuals with bvFTD: mild (CDR Global = 0.5), moderate (CDR Global = 1), and severe (CDR Global > = 2).

### 2.6 Statistical analysis

#### 2.6.1 Group differences

Group differences in covariance between receptor density and structural degeneration were assessed using ANCOVA with group (bvFTD vs. CU) as the independent variable and spatial correlation coefficients as the dependent variable, adjusting for sex. At the group level, bvFTD and CU structural degeneration maps were also compared with each receptor density map using Pearsons’s correlations. Statistical significance was evaluated using spin-based spatial permutation tests (i.e., spin test) to account for spatial autocorrelation (Alexander-Bloch et al., 2018). Spin tests were conducted in R using the “rotate.parcellation” and “perm.sphere.p” algorithms in R (Váša et al., 2018), which generate null distributions by rotating cortical parcellations on a spherical surface. Specifically, 10,000 spherical rotations of the Schaefer 100 parcellation were performed, and permuted *p*-values (*p*_spin_) were derived by comparing the empirically observed Pearson’s correlations between structural degeneration and receptor density maps to the null distribution.

#### 2.6.2 Associations with disease stage and severity

The effect of categorical disease stage on spatial covariance of receptor density and structural degeneration was evaluated using ANCOVA with disease stage as the independent variable and spatial correlation coefficients as the dependent variable, adjusting for sex. Additionally, for each disease stage, average structural degeneration maps were computed and compared with each receptor density map using Pearson’s correlation and statistical significance using spin tests. Linear regression was used to examine the association between disease severity and spatial covariance of receptor density and structural degeneration, adjusting for sex.

To further investigate the relationship between mGluR5 covariance and disease stage, we tested whether patterns of structural degeneration track the mGluR5 density gradient in individuals with bvFTD. Brain regions were ranked according to mGluR5 density (1 = highest density) and degree of structural degeneration (1 = greatest volume difference between bvFTD and CU). Spearman correlations were used to test associations between regional ranks. This process was repeated within each disease stage to determine how the strength of association changes with advancing disease stage.

#### 2.6.3 Associations with clinical outcomes

Linear regression was used to examine the association between spatial covariance of receptor density and structural degeneration and executive function (i.e., letter- and category-guided fluency, DSB), neuropsychiatric symptoms and visuospatial function, adjusting for sex and education.

#### 2.6.4 Correction for multiple comparisons

Tests for each receptor were subjected to Bonferroni correction, with statistical significance set at a corrected alpha level of *p* < .0056 (0.05/9 tests). Reported *p*-values are Bonferroni adjusted (*p_adj_*) with the exception of permuted *p*-values (*p*_spin_).

#### 2.6.5 Dominance analysis

To assess the strength of effects for each spatial covariance of receptor density map and structural degeneration on group differences, disease stage and severity, and clinical outcomes, we conducted dominance tests. First, a linear model including all spatial covariance predictors was fit, then the contribution of each predicted to the model fit was calculated. We report the relative dominance of predictors, that is the R^2^ contributed by the predictor as a percentage of the model R^2^.

#### 2.6.6 Supplementary sensitivity analyses

Analyses stratifying by confirmed pathogenic genetic variant status (Supplementary Table S2), genetic or pathology-confirmed proteinopathy (Supplementary Table S3), and sex (Supplementary Table S4) are reported in the supplement. Analyses controlling for global atrophy are reported in Supplementary Table S5.

## 3 Results

### 3.1 Descriptive statistics

**Fig. 1.**
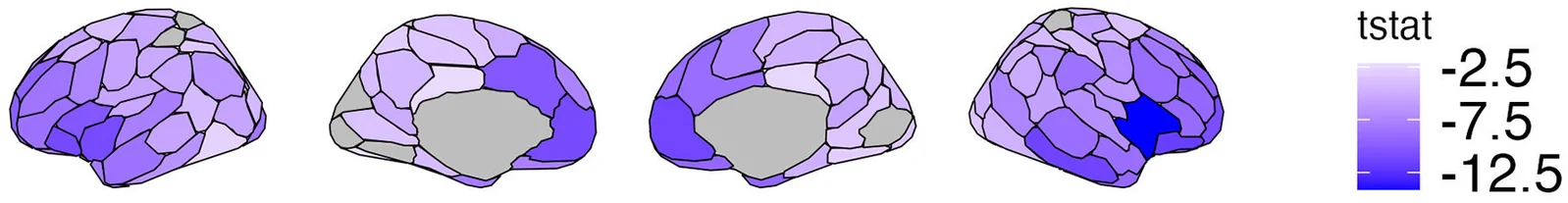
Structural degeneration map. T-statistics of volume differences between individuals with bvFTD and CU after thresholding by FDR-adjusted *p*-values (*q *< .05) for the Schaefer 100 x 7 parcellation.

The bvFTD and CU groups did not differ in age at MRI or education. CU participants were more likely to be female and to have undergone MRI protocol 1. Descriptive statistics for clinical measures are reported in Table 1. Figure 1 demonstrates that, compared with CU, individuals with bvFTD had greater structural degeneration across most brain regions, with the most pronounced degeneration observed in the frontal and temporal regions as expected. Receptor maps are reported in Hansen et al. (2022). All maps were significantly correlated with each other: mGluR5 and 5HT-6 is *r* = 0.71 (*p* < .001), mGluR5 and VAChT is *r* = 0.53 (*p* < .001), mGluR5 and NMDA is *r* = 0.46 (*p* < .001), NMDA and 5HT-6 is *r* = 0.53 (*p* < .001), NMDA and VAChT is *r* = 0.51 (*p* < .001), and 5-HT6 and VAChT is *r* = 0.35 (*p* < .001).

### 3.2 Spatial covariance of structural degeneration and receptor maps: Group differences

**Fig. 2.**
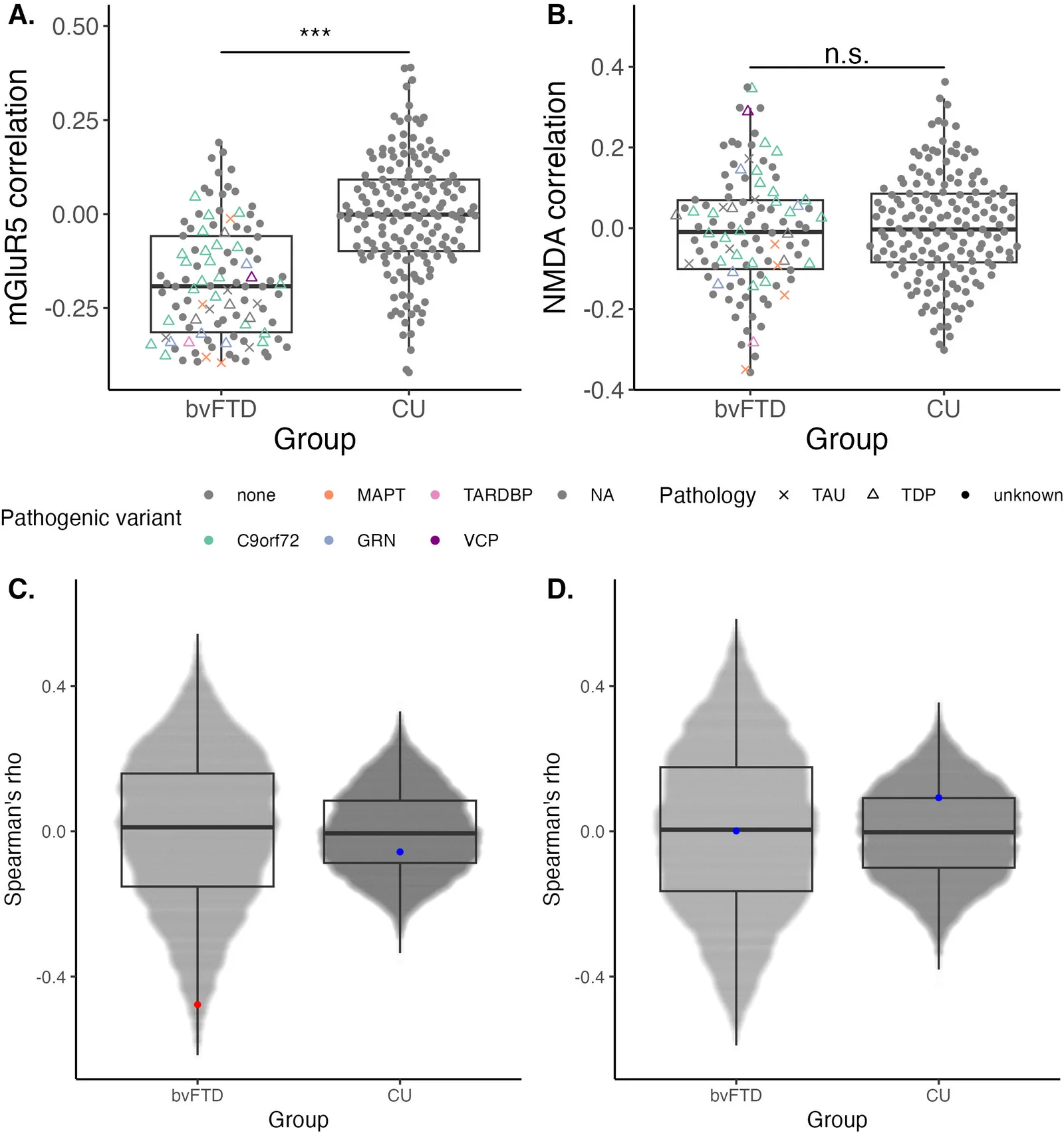
Group differences in spatial covariance of structural degeneration and receptor maps. (A, B) Boxplots display group differences in receptor covariance tested with ANCOVA. Individuals with bvFTD have stronger covariance of structural degeneration and mGluR5 (A) but not NMDA (B) density compared with CU. Among the bvFTD group, autopsy or genetically confirmed pathology is indicated by datapoint shape, and pathogenic genetic variant is indicated by datapoint color. (C, D) Group comparisons are replicated using spin-based permutation tests. Black dots represent the spin-based null distribution. Colored dots indicate empirical rho values with red reflecting *p*_spin_ < = .05 and blue reflecting non-significant *p*_spin_.

Spatial covariance of mGluR5 receptor density and structural degeneration (F(1, 268) = 84.11, *p_adj_* < .001, η_p_^2^ = .24) significantly differed by group (Fig. 2). Across all parcellations (see Supplementary Fig. S1), individuals with bvFTD showed greater mGluR5 ~ structural degeneration covariance indicating that regions with greater degeneration also had a greater density of mGluR5. Group differences in NMDA covariance were not observed.

### 3.3 Spatial covariance of structural degeneration and receptor maps: Associations with disease stage and severity

**Fig. 3.**
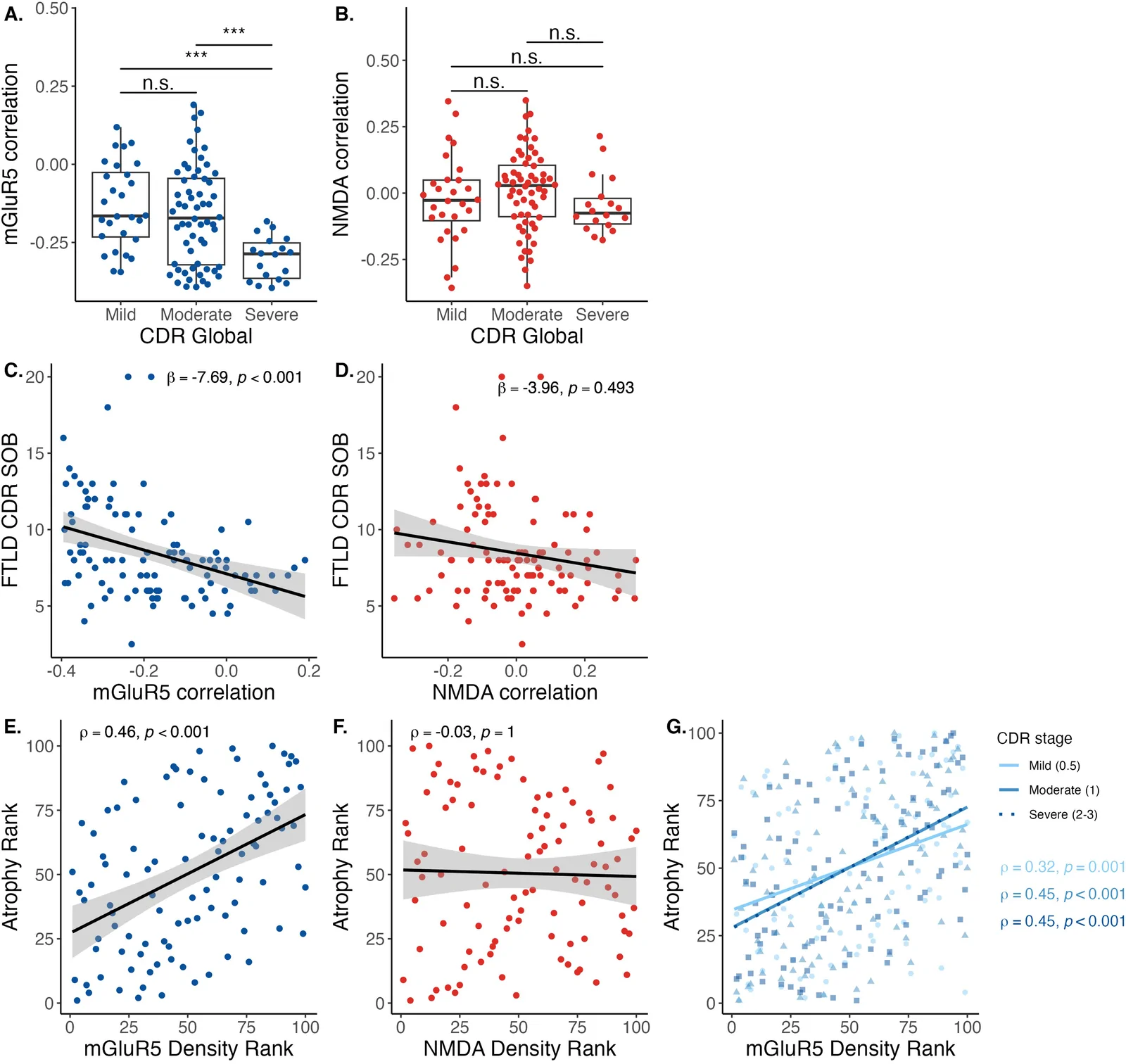
Association of disease stage and severity and spatial covariance of structural degeneration and receptor maps. (A, B) Boxplots display differences in receptor spatial covariance by disease stage. Pairwise comparisons were calculated with t-tests and FDR correction. Increasing disease stage is associated with stronger correlation of structural degeneration and mGluR5 (A; blue) but not NMDA (B; red) density. (C, D) Scatter plots show linear regression lines for associations between receptor spatial covariance and disease severity. Increasing disease severity is associated with stronger covariance of structural degeneration and mGluR5 (C; blue) but not NMDA (D; red) density. (E–G) Scatter plots show Spearman’s correlations between the rank of regions according to receptor density and atrophy. Rank of mGluR5 (E) but not NMDA (F) is positively correlated with atrophy rank of full bvFTD sample. Rank of mGluR5 is also correlated with atrophy rank within each disease stage (G). ***p < .001.

In bvFTD, greater covariance between mGluR5 and structural degeneration was related to more advanced disease stage (CDR Global; F(2, 102) = 8.54, *p_adj_* = .0033; η_p_^2^ = .14; Fig. 3A) and disease severity (FTLD CDR SOB; β = -7.69; *p_adj_* = .0009; Fig. 3B) for all parcellations (see Supplementary Fig. S2). Covariance of NMDA and structural degeneration was not associated with disease stage or severity (see Fig. 3C, D).

Regional rank of mGluR5 density and atrophy exhibited strong correspondence (ρ = .46, *p_adj_* < .001; Fig. 3E). Moreover, we observed stronger correlations at more advanced disease stages (mild: ρ = .32, *p_unadj_* = .0014; moderate: ρ = .45, *p_unadj_* < .001; severe: ρ = .45, *p_unadj_* < .001; Fig. 3F), suggesting that atrophy tracks the mGluR5 density gradient. Regional rank of NMDA and atrophy was not significantly correlated (Fig. 3G).

### 3.4 Spatial covariance of structural degeneration and receptor maps: Associations with clinical outcomes

**Fig. 4.**
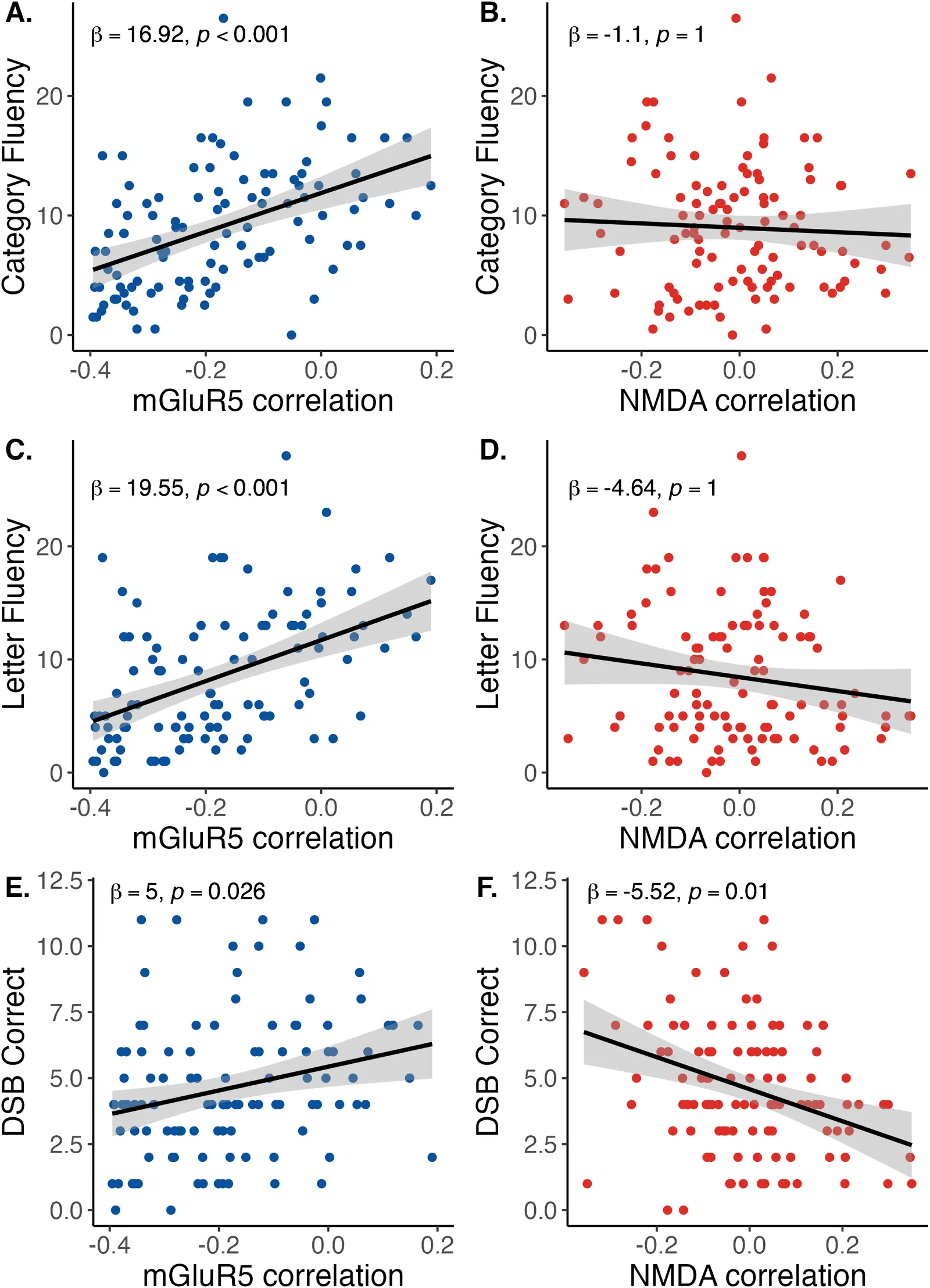
Association of clinical outcomes and spatial covariance of glutamatergic receptors and structural degeneration. (A–F) Scatter plots show linear regression lines for associations between receptor spatial covariance and executive function measures. Worse category-guided fluency (A, B) and letter-guided fluency (C, D) were associated with stronger covariance of structural degeneration and mGluR5 (A, C) but not NMDA (B, D) density. For digit span backward (DSB) number of correct trials (E, F), stronger covariance of structural degeneration and mGluR5 (E) was associated with worse performance and NMDA (F) was associated with better performance.

As the strength of association between structural degeneration and higher mGluR5 density increased, scores on tests of executive function, but not visuospatial function or neuropsychiatric symptom severity, worsened. Specifically, higher covariance of mGluR5 and structural degeneration (see Supplementary Table S6 and Fig. 4) was related to worse DSB (β = 5.0; *p_adj_* = .0257), category-guided fluency (β = 17; *p_adj_* < .001), and letter-guided fluency (β = 20; *p_adj_* < .001) but not NPI severity or Benson Figure Copy, across parcellations (see Supplementary Fig. S3). Greater covariance of NMDA and structural degeneration (see Supplementary Table S7 and Fig. 4) was related to better performance on DSB (β = -5.5; *p_adj_* = .0101), suggesting that co-localization of high NMDA density and greater structural degeneration is associated with better working memory. However, this finding did not replicate across parcellations (see Supplementary Fig. S4). NMDA covariance was not associated with category- and letter-guided fluency, NPI severity, or Benson Figure Copy.

### 3.5 Sensitivity analyses

#### 3.5.1 Effects of mGluR5 with NMDA

To further explore the pattern of results for glutamatergic receptors, we ran an additional series of linear regressions including spatial covariance metrics for both mGluR5 and NMDA as predictors of clinical outcomes. Given the exploratory nature of these regressions, *p*-values were not corrected for multiple comparisons. Of note, the mGluR5 and NMDA maps were moderately correlated with each other (*r* = 0.46) and spatial covariance of mGluR5 and NMDA with structural degeneration were weakly correlated (*r* = 0.21). These analyses confirmed the importance of spatial covariance of mGluR5 in predicting clinical features of bvFTD (see Table 2). NMDA remained a predictor of DSB (β = -6.7; *p* < .001). As shown in Figure 4, mGluR5 (Fig. 4E) and NMDA (Fig. 4F) spatial covariance with structural degeneration have opposing effects where negative mGluR5, but positive NMDA, correlation with structural degeneration is related to worse performance on a measure of working memory.

**Table 2.** Associations of spatial covariance of structural degeneration and glutamatergic receptor maps with clinical outcomes.

Schaefer 100	FTLD CDR SOB	DSB correct	Category fluency	Letter fluency	Figure copy	NPI severity
mGluR5	-7.2*** (-11, -3.3)	6.2*** (3.2, 9.3)	18*** (12, 24)	21*** (15, 27)	5.2* (0.59, 9.8)	2.9 (-4.7, 11)
NMDA	-2.4 (-6.3, 1.5)	-6.7*** (-9.8, -3.6)	-4.4 (-11, 1.7)	-8.6** (-15, -2.2)	-5.2* (-9.9, -0.52)	-0.94 (-8.7, 6.8)
Sex	-0.79 (-2.0, 0.39)	0.67 (-0.26, 1.6)	1.1 (-0.77, 2.9)	1.2 (-0.77, 3.1)	2.0** (0.57, 3.4)	0.47 (-1.9, 2.8)
Education		0.15 (-0.03, 0.33)	0.25 (-0.11, 0.60)	0.48* (0.11, 0.86)	0.01 (-0.26, 0.28)	-0.03 (-0.48, 0.42)
(Intercept)	7.7*** (6.5, 8.9)	2.9* (0.08, 5.7)	7.5** (1.9, 13)	3.8 (-2.0, 9.6)	14*** (9.5, 18)	11** (3.5, 18)
R²	0.163	0.271	0.270	0.350	0.151	0.008

β (95% confidence interval).

Uncorrected *p*-values: *p < .05; **p < 0.01; ***p < 0.001.

Abbreviation: SOB = sum of boxes; DSB = digit span backward; NPI = neuropsychiatric inventory.

#### 3.5.2 Permutation analyses

The group-level average bvFTD structural degeneration map had a moderate negative correlation with the mGluR5 map (*r* = -0.48, *p*_spin_ = .0122; Fig. 2C) and a weak negative correlation with 5HT6 map (*r* = -0.31, *p*_spin_ = .0238) but no significant correlations with the NMDA (Fig. 2D) or VAChT maps. The group-level CU structural map was not correlated with any of the receptor maps. Disease stage average structural degeneration maps were then compared with receptor maps (see Supplementary Fig. S5). In mild bvFTD, no significant correlations emerged. In moderate bvFTD, mGluR5 (*r* = -0.45, *p*_spin_ = .0161) and 5HT6 (*r* = -0.28, *p*_spin_ = .0335) were significant. In severe bvFTD, mGluR5 (*r* = -0.49, *p*_spin_ = .0134), 5HT6 (*r* = -0.33, *p*_spin_ = .0142), and VAChT (*r* = -0.34, *p*_spin_ = .0423) were significant. NMDA was non-significant across all stages.

#### 3.5.3 Non-glutamatergic receptors: Group differences

Spatial covariance of VAChT (F(1, 268) = 33.93, *p_adj_* < .001, η_p_^2^ = .11) and 5HT6 (F(1, 268) = 75.89, *p_adj_* < .001, η_p_^2^ = .22) receptor density with structural degeneration differed by group, with individuals with bvFTD showing greater covariance compared with CU.

#### 3.5.4 Non-glutamatergic receptors: Disease stage and severity

Covariance of VAChT and structural degeneration was related to greater disease severity (FTLD CDR SOB; β = -6.1; *p_adj_* = .0189) but not more advanced disease stages (see Supplementary Table S8). Covariance of 5HT6 and structural degeneration was related to more advanced disease stages (CDR Global; F(2, 102) = 6.42, *p_adj_* = .0212; η_p_^2^ = .11) and greater disease severity (FTLD CDR SOB; β = -7.6; *p_adj_* = .00582) (see Supplementary Table S9). Regional rank of VAChT (ρ = .29, *p_adj_* = .0364) but not 5HT6 receptor maps and atrophy were significantly correlated.

#### 3.5.5 Non-glutamatergic receptors: Clinical outcomes

Greater overlap of degenerated regions and both high VAChT and 5HT6 density is associated with worse executive function. Specifically, covariance of VAChT and structural degeneration was related to category-guided (β = 9.9; *p_adj_* = .0290) and letter-guided (β = 11; *p_adj_* = .0215) fluency, but these associations did not replicate across all parcellations (see Supplementary Table S8). VAChT covariance was not associated with DSB, Benson Figure Copy, or NPI severity. The same pattern of associations was found for covariance of 5HT6 and structural degeneration: greater covariance was associated with worse category-guided (β = 16, *p_adj_* < .001) and letter-guided (β = 16, *p_adj_* < .001) fluency but not DSB, Benson Figure Copy, or NPI severity (see Supplementary Table S9).

#### 3.5.6 Dominance analysis

To assess relative dominance of predictors, we report the R^2^ contributed by the predictor as a percentage of the model R^2^. Disease severity was best predicted by mGluR5 (0.40), followed by 5HT6 (0.24), VAChT (0.21) and NMDA (0.07). DSB was best predicted by NMDA (0.52), followed by mGluR5 (0.28), 5HT6 (0.07), and VAChT (0.02). Category fluency was best predicted by mGluR5 (0.46), 5HT6 (0.26), VAChT (0.12), and NMDA (0.09). Letter-guided fluency was best predicted by mGluR5 (0.45), followed by NMDA (0.20), 5HT6 (0.18), and VAChT (0.11).

## 4 Discussion

The current study leverages a large sample of individuals with bvFTD to examine the relationship between cortical glutamate receptor distribution with structural degeneration and clinical features of bvFTD. Consistent with past work demonstrating lower glutamate levels in bvFTD and lower mGluR5 availability, we found that cortical structural degeneration is greater in regions that have a high density of mGluR5 in bvFTD but not CU participants. We also present evidence from cross-sectional data that structural degeneration at each disease stage tracks with mGluR5 density, suggesting a stage-dependent relationship between degeneration and the mGluR5 density gradient. Moreover, in individuals with bvFTD, the extent to which high mGluR5 density covaried with greater structural degeneration was associated with more advanced disease stages and severity and worse executive functioning. Together, findings establish the association of a component of the glutamate system, mGluR5 density, with clinical features of bvFTD in a novel manner.

The current study provides independent and converging evidence of the importance of glutamate, particularly mGluR5, in bvFTD. A PET study demonstrated that mGluR5 availability is significantly lower in bvFTD than in CU (Leuzy et al., 2016), however, this study was conducted in a sample of five bvFTD participants and did not associate mGluR5 availability with clinical outcomes. Of note, prior studies have employed a similar approach to understand the role of neurotransmitter receptors/transporters, including mGluR5, in bvFTD and do not find an effect of mGluR5 (Bi et al., 2026; Morais et al., 2025). This is surprising given the current study’s findings but could be due to smaller samples sizes, differences in imaging modalities (e.g., Bi et al. (2026) measured glucose metabolism), cohort characteristics (e.g., Bi et al. (2026) combined svPPA and bvFTD into one FTD group), and methodological choices. Indeed, although Morais et al. (2025) also examined GMV, they examined correlations between GMV adjusted for ICV, whereas in the current study, structural degeneration was assessed by w-scores. W-scores facilitate inferring atrophy by comparing bvFTD participants’ brain volumes with CU participants with similar characteristics (e.g., same age, ICV, and MRI protocol). Current findings add to this body of work, demonstrating that the extent to which mGluR5 density and structural degeneration maps are spatially correlated aligns with both disease severity and executive dysfunction.

A link between reduced mGluR5 and executive dysfunction would have important clinical implications, as mGluR5 is known to contribute to cognitive functioning via interactions with NMDAs in long-term potentiation (J.-H. Kim et al., 2020; Wang et al., 2015). Our findings suggest that disruption of mGluR5 may have a role in the severity, and perhaps emergence, of executive dysfunction in bvFTD. In turn, these associations indicate that measures of executive dysfunction may be useful clinical outcomes when testing interventions that target mGluR5 function. Due to evidence of mGluR5 disruption for a number of neuropsychiatric disorders and the favorable properties of mGluR5 (e.g., mGluR5 can be visualized with PET), several compounds to modulate mGluR5 activity are under development or investigation (Pillai & Tipre, 2016). Thus, current findings represent an important step in motivating data-driven assessment of glutamatergic interventions to potentially alleviate symptoms of bvFTD. Concurrent with such investigations, it will also be important to characterize the molecular mechanisms of observed associations between mGluR5, structural degeneration, and clinical outcomes. mGluR5 is a key regulator of glutamatergic neurotransmission, synaptic plasticity, and modulation of microglial and astrocytic responses (Benarroch, 2008; Budgett et al., 2022; J. Kim et al., 2025). Thus, in addition to its role in executive functions, mGluR5 may also contribute to downstream neurodegenerative processes through facilitation of an excitotoxic environment (J. Kim et al., 2025; Leuzy et al., 2016). In turn, disruption of mGluR5-mediated signaling in the same regions where structural degeneration is also observed in bvFTD may contribute to executive dysfunction characteristic of bvFTD. Although our cross-sectional findings do not establish causality, the spatial covariance of mGluR5 density and structural degeneration and associations with executive dysfunction are consistent with the possibility that mGluR5-related processes are linked to clinically relevant disease mechanisms.

We did not observe a relationship between covariance of mGluR5 and structural degeneration with neuropsychiatric symptom severity. This result could be due to limitations of our measure of neuropsychiatric symptom severity, the NPI, in capturing neuropsychiatric symptoms common in bvFTD. Indeed, the NPI lacks specificity (Lai, 2014) and was developed to capture the neuropsychiatric features common in AD (Cummings, 1997). In turn, the NPI does not measure key behavioral symptoms of bvFTD, including loss of empathy and social comportment (Jiskoot et al., 2023). Future work should examine associations with additional behavioral measures that assess the neuropsychiatric symptoms observed in bvFTD, particularly loss of empathy and social comportment, two features that are also linked to frontal cortices.

Spatial covariance of NMDA and structural degeneration maps did not differ by group (bvFTD vs. CU) or disease stage (mild, moderate, severe). This finding is consistent with past work examining post-mortem tissue from individuals with bvFTD that have found less mGluR5 and AMPA receptors but inconsistent evidence of NMDA changes relative to controls (Bowen et al., 2008; Procter et al., 1999). Although the covariance of NMDA density and structural degeneration did not differ between bvFTD and CU participants, it did predict performance on digit span backward, a measure of working memory, particularly when covariance of mGluR5 and structural degeneration was also included in the model as a predictor. Of note, this association was only observed for the Schaefer 100 parcellation and was not replicated for additional parcellations reported in the Supplement. Of note, this association was only observed for the Schaefer 100 and Lausanne parcellations and was not replicated for additional parcellations reported in Supplementary Table S7. That is, working memory was related to both (i) co-localization of high mGluR5 density and high structural degeneration and (ii) co-localization of high NMDA density and low structural degeneration. The association of NMDA covariance with working memory is contrary to what would be predicted, given the documented positive role of NMDA activation in learning and memory (Lüscher & Malenka, 2012). NMDA covariance did not differ between individuals with bvFTD relative to CU, nor between disease stages, making this result difficult to interpret. Moreover, this finding did not replicate across parcellations, and thus, this exploratory result requires replication and further investigation. A few methodological factors may also contribute to this result. Unlike the mGluR5 map that reflects receptor availability, the NMDA map is derived from a tracer that binds to open (i.e., active) receptors, and thus, reflects activation/use-dependent signal. Additionally, the NMDA map is derived from a smaller sample and may be less reliable (Hansen et al., 2022). Thus, future work directly measuring NMDA availability in individuals with bvFTD with PET imaging is needed. If supported by future work, the present pattern of findings with regard to NMDA covariance is potentially interesting, given the equivocal effects of NMDA therapy in bvFTD (Boxer et al., 2013; Vercelletto et al., 2011).

Although our study benefitted from a large sample of well-characterized individuals with bvFTD, we are limited by our reliance on an indirect estimation of the impact of disease on glutamate receptors in individuals with bvFTD. An ideal design to test this question would be to acquire *in vivo* mGluR5 PET and glutamate-weighted chemical exchange saturation transfer (GluCEST) in the same bvFTD patients, directly linking each individual’s receptor density to their glutamatergic signal. However, this approach is difficult as PET requires specialized radioligands (e.g., [¹¹C]ABP688 or [^18^F]FPEB) and on-site radiochemistry, carries radiation exposure that constrains repeat imaging, can interrogate only a single target per session, and imposes substantial cost and patient burden. Thus, in the present study, we employed normative receptor maps—a radiation-free, reproducible, and scalable reference—as a first step. We additionally took several steps to evaluate the reliability and specificity of our findings. To assess reliability, we calculated the covariance of mGluR5 with structural degeneration across different resolutions of the functional Schaefer atlases and in an anatomic atlas, the Lausanne atlas. We demonstrate that covariance of mGluR5 and structural degeneration is related to disease stage/severity and clinical outcomes of bvFTD regardless of parcellation and resolution. To assess specificity, we used non-parametric spin tests, which address the possibility that results are driven by spatial autocorrelation (Hansen & Misic, 2025) and we examined covariance of structural degeneration and VAChT, a component of the acetylcholine neurotransmitter system that is not highly impacted in bvFTD (Murley & Rowe, 2018), and of 5HT6, a receptor map highly correlated with mGluR5 (*r* = 0.7). Although the covariance of these receptor maps with structural degeneration shows some of the tested associations, models predicting disease stage/severity and clinical outcomes from covariance of mGluR5 and structural degeneration had stronger effect sizes than models with covariance of VAChT or 5HT6 and structural degeneration as the predictor. We are not asserting that these receptors, especially 5HT6, are not implicated in bvFTD but rather that mGluR5 is particularly important in bvFTD and in the executive features of bvFTD. This assertion—that the effect of mGluR5 covariance with structural degeneration is greater than that of the other maps tests—was further confirmed using dominance analysis.

In addition to the limitation that we did not have a direct measure of glutamate receptors or glutamate level for our entire cohort of individuals with bvFTD, our sample was cross-sectional, limiting conclusions about the disease process, and primarily characterized by clinical syndrome, with only a small subset of individuals with bvFTD characterized by proteinopathy and/or pathogenic genetic variant. Although lower glutamatergic neuron levels in the frontal cortex have been found in both FTLD-TAU and FTLD-TDP-43 (Santillo et al., 2013), there is evidence of interactions between the glutamate cycle and FTLD-TAU (Benussi et al., 2019; Borroni et al., 2017). Indeed, we observed some evidence for this as individuals with bvFTD and confirmed FTLD-TAU appear to demonstrate the strongest association of mGluR5 with structural degeneration (Fig. 1; Supplementary Table S3). Therefore, specific measures of aspects of the glutamatergic system may reveal differences by proteinopathy, which should be examined in other cohorts of individuals with bvFTD and confirmed proteinopathy. Additionally, our sample has a highly homogeneous demographic makeup, with the majority of participants identifying as white and highly educated, limiting generalizability of findings to individuals with bvFTD of all backgrounds. Our findings should be replicated in other cohorts to confirm that identified associations generalize beyond this cohort.

### 4.1 Conclusion

Results demonstrate the importance of a component of the glutamate system, mGluR5 density, in bvFTD in a novel manner through establishing associations between the colocalization of high mGluR5 density and degree of structural degeneration with the severity of disease and core clinical features. We demonstrate preferential structural degeneration in bvFTD in regions of higher mGluR5 density and illustrate significantly higher patterns of covariance in (i) severe compared with mild or moderate dementia, (ii) greater severity of bvFTD symptoms, and (iii) worse cognitive performance, consistent with the hypothesis that the glutamatergic system is vulnerable to neurodegeneration in bvFTD. Future longitudinal analysis and expansion of the current study to measure mGluR5 or glutamate levels *in vivo* could provide additional insights into the natural history of glutamate dysfunction in bvFTD, facilitating hypothesis-driven testing of glutamate-mediating therapies in bvFTD.

## Supplementary Material

Supplementary Material

## Data Availability

The volumetric PET images for receptor density are available at (https://github.com/netneurolab/hansen_receptors). Other datasets used in the current study are available from the corresponding author on reasonable request and approval from the Penn Neurodegenerative Data Sharing Committee. Requests may be submitted using a webform request: https://www.pennbindlab.com/data-sharing.
