# Supplementary material for: Spatial covariance of mGluR5 density and structural degeneration in behavioral variant frontotemporal degeneration
Source: Imaging Neurosci (Camb). 2026 Sep 10;4:IMAG.a.1358. doi: 10.1162/IMAG.a.1358 (PMC13563532; doi:10.1162/IMAG.a.1358)
Supplement: Supplementary file 1 [file IMAG.a.1358_supp.pdf]

Table S1. Summary of studies used to generate normative neurotransmitter maps.

Receptor	Neurotransmitter	Tracer	Measure	n (F)	Age	Reference
mGluR5	Glutamate	[11C]ABP688	BP_{ND}	73 (48)	19.9 ± 3.04	Smart et al.
mGluR5	Glutamate	[11C]ABP688	BP_{ND}	22 (10)	67.9±9.6	Rosa-Neto & Kobayashi
mGluR5	Glutamate	[11C]ABP688	BP_{ND}	28 (13)	33.1 ± 11.2	DuBois et al.
NMDA	Glutamate	[18F]GE-179	V_T	29 (8)	40.9 ± 12.7	Galovic et al.
VAChT	Acetylcholine	[18F]FEOBV	SUVR	4 (1)	37±10.2	Tuominen & Guimond
VAChT	Acetylcholine	[18F]FEOBV	SUVR	18 (13)	66.8 ± 6.8	Aghourian et al.
VAChT	Acetylcholine	[18F]FEOBV	SUVR	5 (1)	68.3 ± 3.1	Bedard et al.
VAChT	Acetylcholine	[18F]FEOBV	SUVR	3 (3)	66.6±0.94	Schmitz & Spreng
5HT6	Serotonin	[11C]GSK215083	BP_{ND}	30 (0)	36.6 ± 9.0	Radhakrishnan et al.

BP_{ND} = non-displaceable binding potential; V_T = tracer distribution volume, SUVR = standard uptake value ratio. Parentheses indicate the number of females in the study sample.

Table S2. Group differences in spatial covariance of receptor density and structural degeneration stratified by confirmed pathogenic variant status.

Receptor	Contrast	Estimate	SE	df	t	p
mGluR5	CU	-0.021	0.014	256	-1.518	0.130
	bvFTD-sporadic	-0.140	0.020	256	-6.933	**0.000**
	bvFTD-C9orf72	-0.129	0.036	256	-3.589	**0.000**
	CU - (bvFTD-sporadic)	0.119	0.027	256	4.427	**0.000**
	CU - (bvFTD-C9orf72)	0.108	0.041	256	2.655	**0.023**
	(bvFTD-sporadic) - (bvFTD-C9orf72)	-0.011	0.038	256	-0.287	0.956
NMDA	CU	0.006	0.012	256	0.532	0.595
	bvFTD-sporadic	-0.017	0.018	256	-0.933	0.352
	bvFTD-C9orf72	0.027	0.032	256	0.832	0.406
	CU - (bvFTD-sporadic)	0.023	0.024	256	0.966	0.599
	CU - (bvFTD-C9orf72)	-0.020	0.037	256	-0.556	0.844
	(bvFTD-sporadic) - (bvFTD-C9orf72)	-0.044	0.034	256	-1.298	0.398
5HT6	CU	-0.013	0.011	256	-1.192	0.234
	bvFTD-sporadic	-0.102	0.016	256	-6.273	**0.000**
	bvFTD-C9orf72	-0.112	0.029	256	-3.862	**0.000**
	CU - (bvFTD-sporadic)	0.089	0.022	256	4.097	**0.000**
	CU - (bvFTD-C9orf72)	0.099	0.033	256	3.004	**0.008**
	(bvFTD-sporadic) - (bvFTD-C9orf72)	0.010	0.030	256	0.329	0.942
VAChT	CU	-0.015	0.014	256	-1.036	0.301
	bvFTD-sporadic	-0.105	0.021	256	-4.917	**0.000**
	bvFTD-C9orf72	-0.088	0.038	256	-2.322	**0.021**
	CU - (bvFTD-sporadic)	0.090	0.028	256	3.160	**0.005**
	CU - (bvFTD-C9orf72)	0.073	0.043	256	1.700	0.207
	(bvFTD-sporadic) - (bvFTD-C9orf72)	-0.017	0.040	256	-0.418	0.908

Estimated marginal means and mean differences for each group and contrast, respectively. Uncorrected p-values are reported; p-values < 0.05 are bolded.

Table S3. Group differences in spatial covariance of receptor density and structural degeneration stratified by genetic or pathology confirmed pathology.

Receptor	Contrast	Estimate	SE	df	t	p
mGluR5	CU	-0.018	0.014	265	-1.317	0.189
	unknown	-0.140	0.020	265	-6.931	**0.000**
	TDP	-0.167	0.030	265	-5.563	**0.000**
	TAU	-0.207	0.056	265	-3.691	**0.000**
	CU - unknown	0.122	0.027	265	4.577	**0.000**
	CU - TDP	0.149	0.036	265	4.173	**0.000**
	CU - TAU	0.188	0.060	265	3.117	**0.011**
	unknown - TDP	0.027	0.033	265	0.804	0.852
	unknown - TAU	0.066	0.056	265	1.177	0.642
	TDP - TAU	0.039	0.059	265	0.675	0.907
NMDA	CU	0.000	0.013	265	0.005	0.996
	unknown	-0.013	0.019	265	-0.704	0.482
	TDP	0.026	0.028	265	0.938	0.349
	TAU	-0.040	0.051	265	-0.780	0.436
	CU - unknown	0.013	0.025	265	0.537	0.950
	CU - TDP	-0.026	0.033	265	-0.787	0.860
	CU - TAU	0.040	0.055	265	0.723	0.888
	unknown - TDP	-0.039	0.031	265	-1.276	0.579
	unknown - TAU	0.027	0.052	265	0.523	0.954
	TDP - TAU	0.066	0.054	265	1.228	0.610
5HT6	CU	-0.012	0.011	265	-1.082	0.280
	unknown	-0.106	0.017	265	-6.379	**0.000**
	TDP	-0.136	0.025	265	-5.519	**0.000**
	TAU	-0.137	0.046	265	-2.978	**0.003**
	CU - unknown	0.094	0.022	265	4.279	**0.000**
	CU - TDP	0.124	0.029	265	4.227	**0.000**
	CU - TAU	0.124	0.050	265	2.510	0.061
	unknown - TDP	0.030	0.027	265	1.101	0.689
	unknown - TAU	0.031	0.046	265	0.667	0.909
	TDP - TAU	0.001	0.048	265	0.015	1.000
VAChT	CU	-0.014	0.015	265	-0.929	0.354
	unknown	-0.103	0.021	265	-4.776	**0.000**
	TDP	-0.108	0.032	265	-3.394	**0.001**
	TAU	-0.173	0.059	265	-2.919	**0.004**
	CU - unknown	0.089	0.028	265	3.142	**0.010**
	CU - TDP	0.095	0.038	265	2.497	0.063
	CU - TAU	0.160	0.064	265	2.491	0.064
	unknown - TDP	0.006	0.035	265	0.158	0.999
	unknown - TAU	0.071	0.060	265	1.186	0.636
	TDP - TAU	0.065	0.062	265	1.051	0.720

Estimated marginal means and mean differences for each group and contrast, respectively. Uncorrected p-values are reported; p-values < 0.05 are bolded.

Table S4. Group differences in spatial covariance of receptor density and structural degeneration stratified by sex.

Receptor	Group	Mean difference	SE	df	t	p
mGluR5	Female	-0.159	0.047	138	-3.413	**0.001**
	Male	-0.125	0.030	127	-4.134	**0.000**
NMDA	Female	-0.010	0.041	138	-0.238	0.812
	Male	-0.018	0.028	127	-0.629	0.531
X5HT6	Female	-0.124	0.036	138	-3.423	**0.001**
	Male	-0.098	0.026	127	-3.706	**0.000**
VAChT	Female	-0.164	0.046	138	-3.588	**0.000**
	Male	-0.074	0.034	127	-2.189	**0.030**

Estimated marginal means and mean differences for each group and contrast, respectively. Uncorrected p-values are reported; p-values < 0.05 are bolded.

Table S5. Results of main analyses including global atrophy as a covariate for Schaefer 100 x 7

**ANCOVA**	**mGluR5**	**NMDA**	**ANCOVA**	**mGluR5**	**NMDA**
Phenotype	86.73***	0.4022	Global CDR	9.412***	1.088
Global atrophy	9.348**	0. 5907	Global atrophy	11.37**	0.0163
Sex	0.7515	0.9716	Sex	0.0002	0.6907

**Regression**	**FTLD CDR SOB**	**DSB Correct**	**Category fluency**	**Letter fluency**	**Figure copy**	**NPI Severity**
mGluR5	-5.7**	2.1	11***	14***	1.9	2.1
	(-9.7,-1.7)	(-1.2,5.4)	(4.9,17)	(7.3,20)	(-3.1,6.9)	(-6.2,10)
Global atrophy	-0.59*	0.83***	1.7***	1.6***	0.68*	0.19
	(-1.1,-0.11)	(0.43,1.2)	(1.0,2.4)	(0.84,2.4)	(0.08,1.3)	(-0.79,1.2)
Sex	-0.44	0.47	0.46	0.68	1.8*	0.41
	(-1.6,0.72)	(-0.47,1.4)	(-1.2,2.2)	(-1.2,2.6)	(0.38,3.2)	(-2.0,2.8)
Education		0.15	0.17	0.45*	0.01	-0.03
		(-0.03,0.33)	(-0.15,0.50)	(0.10,0.81)	(-0.26,0.28)	(-0.48,0.42)
(Intercept)	6.6***	3.9*	11***	6.5*	15***	11**
	(5.2,8.0)	(0.95,6.9)	(6.0,17)	(0.69,12)	(10,19)	(3.5,18)
R^2	0.197	0.265	0.395	0.404	0.154	0.009
NMDA	-3.7	-6.0***	-2.1	-5.7	-4.6*	-0.52
	(-7.5,0.15)	(-8.8,-3.1)	(-8.0,3.7)	(-12,0.77)	(-9.1,-0.06)	(-8.2,7.1)
Global atrophy	-0.85***	0.97***	2.3***	2.3***	0.80**	0.30
	(-1.3,-0.40)	(0.63,1.3)	(1.6,3.0)	(1.6,3.1)	(0.27,1.3)	(-0.60,1.2)
Sex	-0.47	0.34	0.38	0.50	1.7*	0.39
	(-1.7,0.72)	(-0.54,1.2)	(-1.4,2.2)	(-1.5,2.5)	(0.30,3.1)	(-2.0,2.8)
Education		0.07	0.02	0.23	-0.06	-0.06
		(-0.10,0.23)	(-0.32,0.37)	(-0.14,0.61)	(-0.33,0.20)	(-0.51,0.38)
(Intercept)	7.1***	5.2***	13***	9.0**	16***	11**
	(5.7,8.5)	(2.4,8.0)	(7.1,19)	(2.7,15)	(11,20)	(3.7,19)
R^2	0.164	0.362	0.321	0.320	0.182	0.007

Top panel displays ANCOVA results for differences in covariance by group (left) and Global CDR (right) when controlling for global atrophy. Bottom panels show associations of clinical features and spatial covariance of structural degeneration and either mGluR5 or NMDA.

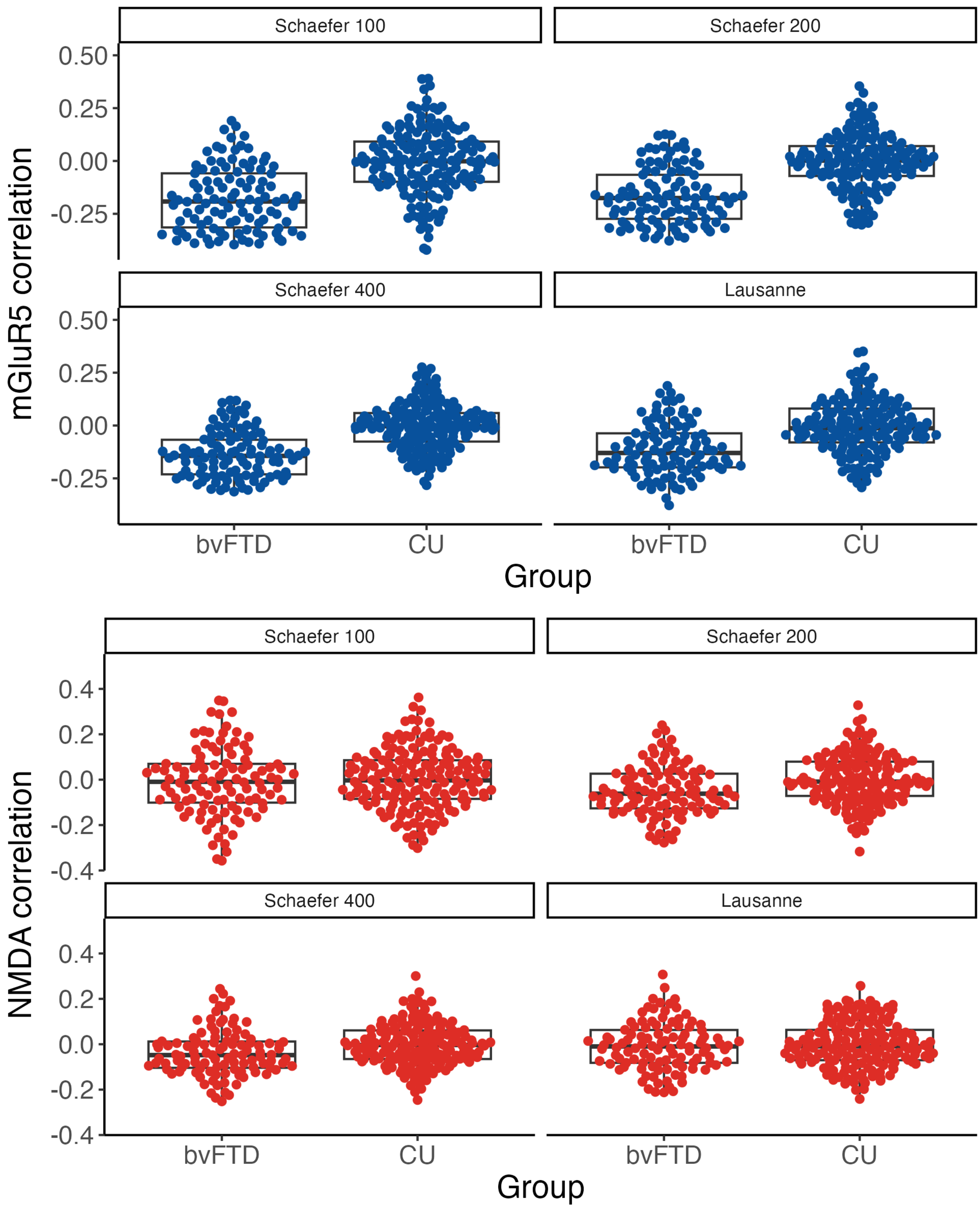

Figure S1. Group differences in covariance of receptor maps and structural degeneration for each parcellation. mGluR5 covariance shown in blue. NMDA covariance shown in red.

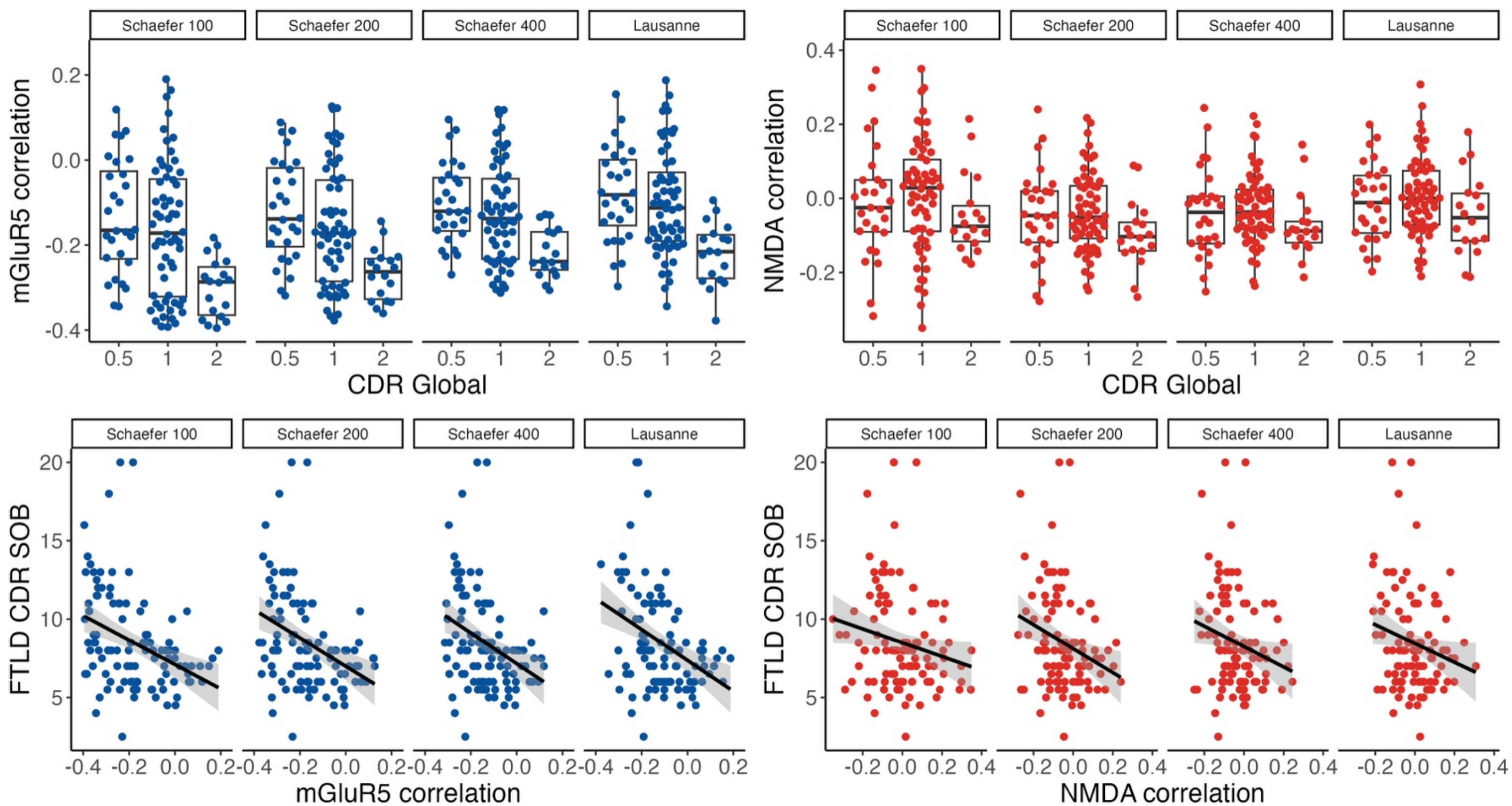

Figure S2. Association of disease stage and severity and covariance of receptor maps and structural degeneration for each parcellation. mGluR5 covariance shown in blue. NMDA covariance shown in red. Disease stage indexed by CDR Global (0.5=mild, 1=moderate, 2=severe). Disease severity indexed by FTLD CDR SOB (higher reflects greater severity).

Table S6. Association of clinical features and spatial covariance of mGluR5 density and structural degeneration for each parcellation.

	Predictor	FTLD CDR SOB	DSB Correct	Category fluency	Letter fluency	Figure copy	NPI Severity
Schaefer 100	mGluR5	-7.7*** (-11,-3.9)	5.0** (1.8,8.2)	17*** (11,23)	20*** (13,26)	4.3 (-0.36,8.9)	2.8 (-4.7,10)
	Sex	-0.72 (-1.9,0.45)	0.82 (-0.19,1.8)	1.2 (-0.68,3.0)	1.3 (-0.64,3.3)	2.1** (0.65,3.5)	0.49 (-1.8,2.8)
	Education		0.21* (0.02,0.40)	0.29 (-0.07,0.64)	0.56** (0.18,0.94)	0.05 (-0.22,0.32)	-0.02 (-0.46,0.42)
	(Intercept)	7.6*** (6.4,8.7)	1.7 (-1.2,4.7)	6.8* (1.3,12)	2.3 (-3.6,8.2)	13*** (8.6,17)	10** (3.5,17)
	R^2	0.151	0.138	0.255	0.304	0.111	0.008
Schaefer 200	mGluR5	-8.9*** (-13,-4.6)	8.5*** (5.0,12)	21*** (14,27)	23*** (16,30)	6.2* (1.0,11)	2.0 (-6.5,10)
	Sex	-0.71 (-1.9,0.45)	0.79 (-0.16,1.7)	1.2 (-0.60,3.0)	1.4 (-0.57,3.3)	2.1** (0.66,3.5)	0.51 (-1.8,2.8)
	Education		0.20* (0.02,0.38)	0.23 (-0.11,0.57)	0.49* (0.12,0.86)	0.04 (-0.23,0.31)	-0.04 (-0.48,0.40)
	(Intercept)	7.5*** (6.3,8.7)	2.3 (-0.49,5.2)	8.1** (2.7,13)	3.8 (-2.1,9.6)	13*** (9.0,17)	10** (3.5,17)
	R^2	0.154	0.237	0.293	0.326	0.129	0.004
Schaefer 400	mGluR5	-9.7*** (-15,-4.5)	10*** (5.9,14)	24*** (16,32)	27*** (18,36)	6.9* (0.72,13)	4.4 (-5.8,15)
	Sex	-0.75 (-1.9,0.44)	0.82 (-0.13,1.8)	1.3 (-0.54,3.1)	1.5 (-0.49,3.4)	2.1** (0.69,3.5)	0.50 (-1.8,2.8)
	Education		0.20* (0.02,0.38)	0.22 (-0.13,0.56)	0.48* (0.10,0.85)	0.04 (-0.23,0.31)	-0.03 (-0.47,0.41)
	(Intercept)	7.6*** (6.5,8.8)	2.3 (-0.50,5.2)	8.1** (2.6,13)	3.7 (-2.1,9.6)	13*** (9.0,17)	11** (3.7,18)
	R^2	0.129	0.233	0.283	0.313	0.123	0.009
Lausanne	mGluR5	-9.7*** (-14,-5.1)	8.3*** (4.6,12)	21*** (14,28)	25*** (17,32)	7.0* (1.5,13)	3.6 (-5.4,13)
	Sex	-0.72 (-1.9,0.44)	0.83 (-0.14,1.8)	1.3 (-0.54,3.1)	1.5 (-0.47,3.4)	2.1** (0.69,3.5)	0.51 (-1.8,2.8)
	Education		0.17 (-0.01,0.36)	0.16 (-0.19,0.50)	0.41* (0.05,0.78)	0.02 (-0.24,0.29)	-0.04 (-0.48,0.39)
	(Intercept)	7.8*** (6.8,8.9)	2.3 (-0.57,5.2)	8.1** (2.6,14)	3.9 (-1.9,9.7)	13*** (9.1,18)	11** (3.7,18)
	R^2	0.159	0.209	0.273	0.329	0.135	0.008

β (95% confidence interval) *p<0.05; **p<0.01; ***p<0.001; Abbreviation: SOB = Sum of boxes; DSB = Digit span backwards; NPI = Neuropsychiatric Inventory

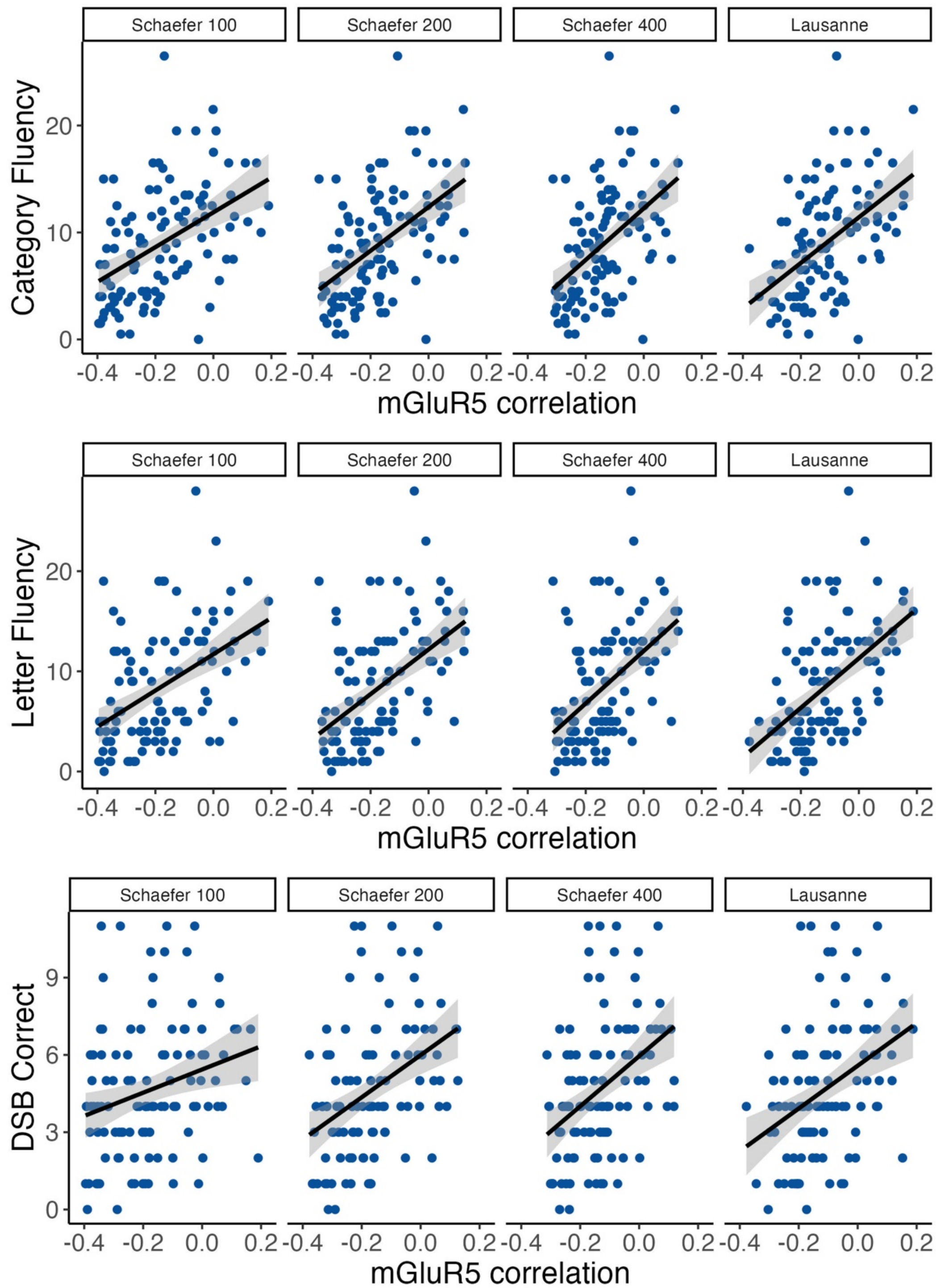

Figure S3. Association of clinical features and spatial covariance of mGluR5 density and structural degeneration for each parcellation.

Table S7. Association of clinical features and spatial covariance of NMDA density and structural degeneration for each parcellation.

	Predictor	FTLD CDR SOB	DSB Correct	Category fluency	Letter fluency	Figure copy	NPI Severity
Schaefer 100	NMDA	-4.0 (-8.0,0.08)	-5.5** (-8.8,-2.3)	-1.1 (-8.0,5.9)	-4.6 (-12,2.9)	-4.2 (-8.9,0.46)	-0.39 (-8.0,7.2)
	Sex	-0.91 (-2.2,0.33)	0.82 (-0.18,1.8)	1.5 (-0.63,3.6)	1.6 (-0.65,3.9)	2.1** (0.66,3.5)	0.54 (-1.8,2.9)
	Education		0.10 (-0.09,0.29)	0.10 (-0.30,0.51)	0.31 (-0.13,0.75)	-0.04 (-0.31,0.24)	-0.05 (-0.50,0.39)
	(Intercept)	9.0*** (8.0,10)	2.5 (-0.49,5.5)	6.5* (0.05,13)	2.5 (-4.4,9.5)	13*** (9.1,18)	10** (3.3,17)
	R^2	0.050	0.153	0.026	0.067	0.109	0.002
Schaefer 200	NMDA	-7.7** (-13,-2.4)	-4.4 (-8.9,0.21)	5.7 (-3.7,15)	1.7 (-8.6,12)	-4.0 (-10,2.4)	-2.5 (-13,7.7)
	Sex	-0.85 (-2.1,0.37)	0.92 (-0.11,2.0)	1.5 (-0.61,3.6)	1.7 (-0.59,4.0)	2.2** (0.74,3.6)	0.55 (-1.8,2.9)
	Education		0.12 (-0.08,0.32)	0.16 (-0.24,0.56)	0.37 (-0.07,0.82)	-0.03 (-0.30,0.25)	-0.07 (-0.52,0.37)
	(Intercept)	8.6*** (7.7,9.6)	2.0 (-1.1,5.0)	5.8 (-0.51,12)	1.6 (-5.3,8.5)	13*** (8.7,17)	10** (3.5,17)
	R^2	0.088	0.091	0.039	0.054	0.095	0.005
Schaefer 400	NMDA	-6.8* (-13,-0.86)	-4.4 (-9.4,0.70)	2.7 (-7.6,13)	-0.64 (-12,11)	-3.6 (-11,3.4)	-2.4 (-14,8.9)
	Sex	-0.90 (-2.1,0.34)	0.89 (-0.15,1.9)	1.5 (-0.59,3.6)	1.7 (-0.59,4.0)	2.1** (0.70,3.6)	0.53 (-1.8,2.8)
	Education		0.12 (-0.08,0.32)	0.14 (-0.27,0.54)	0.35 (-0.09,0.80)	-0.02 (-0.30,0.25)	-0.07 (-0.52,0.38)
	(Intercept)	8.8*** (7.8,9.8)	2.0 (-1.1,5.2)	6.0 (-0.39,12)	1.8 (-5.1,8.8)	13*** (8.7,17)	11** (3.5,18)
	R^2	0.062	0.085	0.028	0.053	0.091	0.004
Lausanne	NMDA	-5.8* (-11,-0.14)	-6.2* (-11,-1.5)	0.87 (-9.0,11)	0.11 (-11,11)	-2.8 (-9.5,3.9)	-3.2 (-14,7.6)
	Sex	-0.73 (-2.0,0.51)	1.0* (0.02,2.1)	1.5 (-0.64,3.6)	1.7 (-0.60,4.0)	2.2** (0.77,3.7)	0.61 (-1.7,2.9)
	Education		0.10 (-0.10,0.29)	0.12 (-0.29,0.53)	0.36 (-0.09,0.81)	-0.02 (-0.30,0.26)	-0.08 (-0.53,0.37)
	(Intercept)	8.9*** (7.9,9.9)	2.4 (-0.67,5.5)	6.2 (-0.27,13)	1.7 (-5.3,8.7)	13*** (8.7,17)	11** (3.7,18)
	R^2	0.053	0.118	0.025	0.053	0.088	0.006

β (95% confidence interval) *p<0.05; **p<0.01; ***p<0.001; Abbreviation: SOB = Sum of boxes; DSB = Digit span backwards; NPI = Neuropsychiatric Inventory

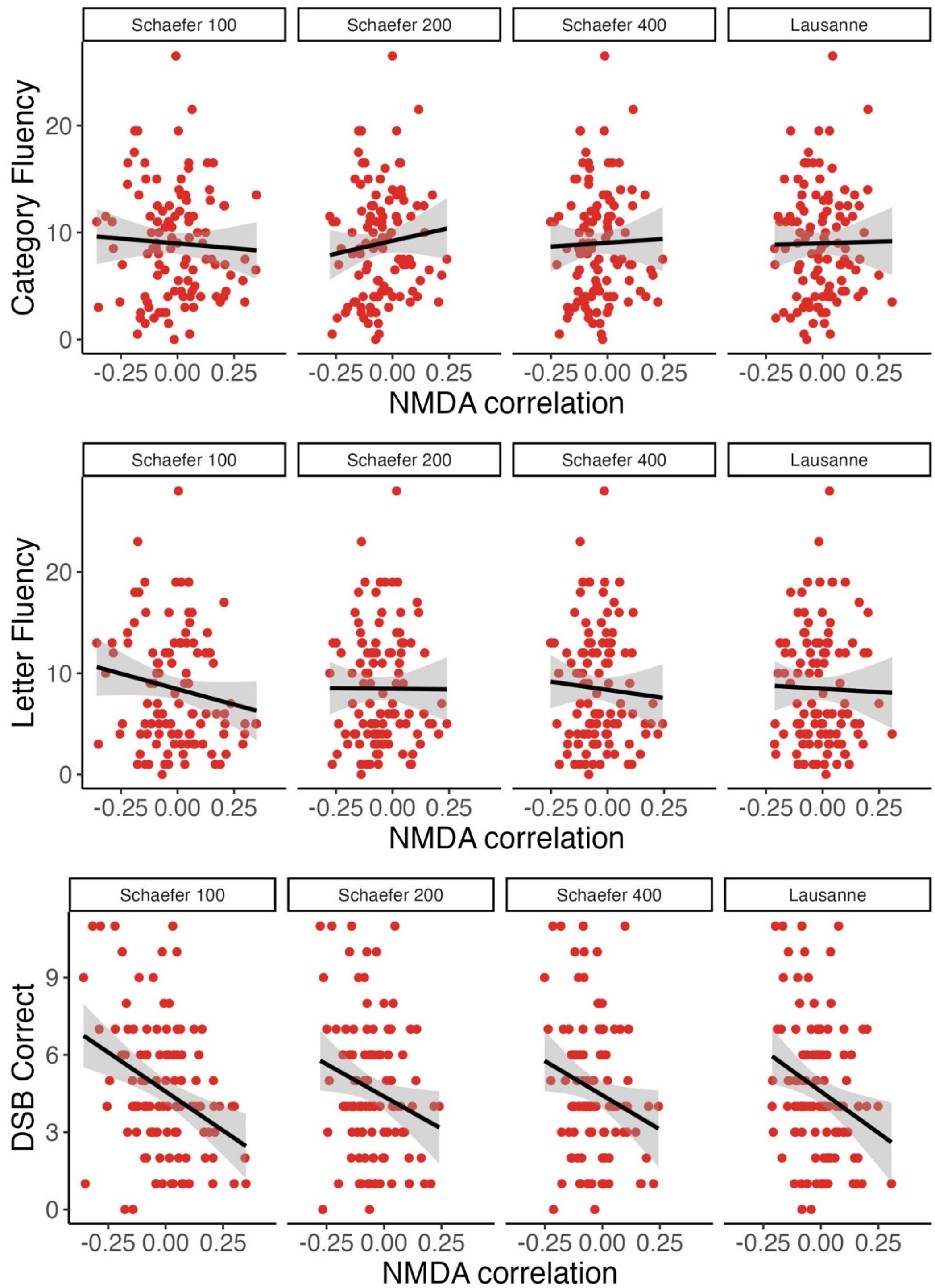

Figure S4. Association of clinical features and spatial covariance of NMDA density and structural degeneration for each parcellation.

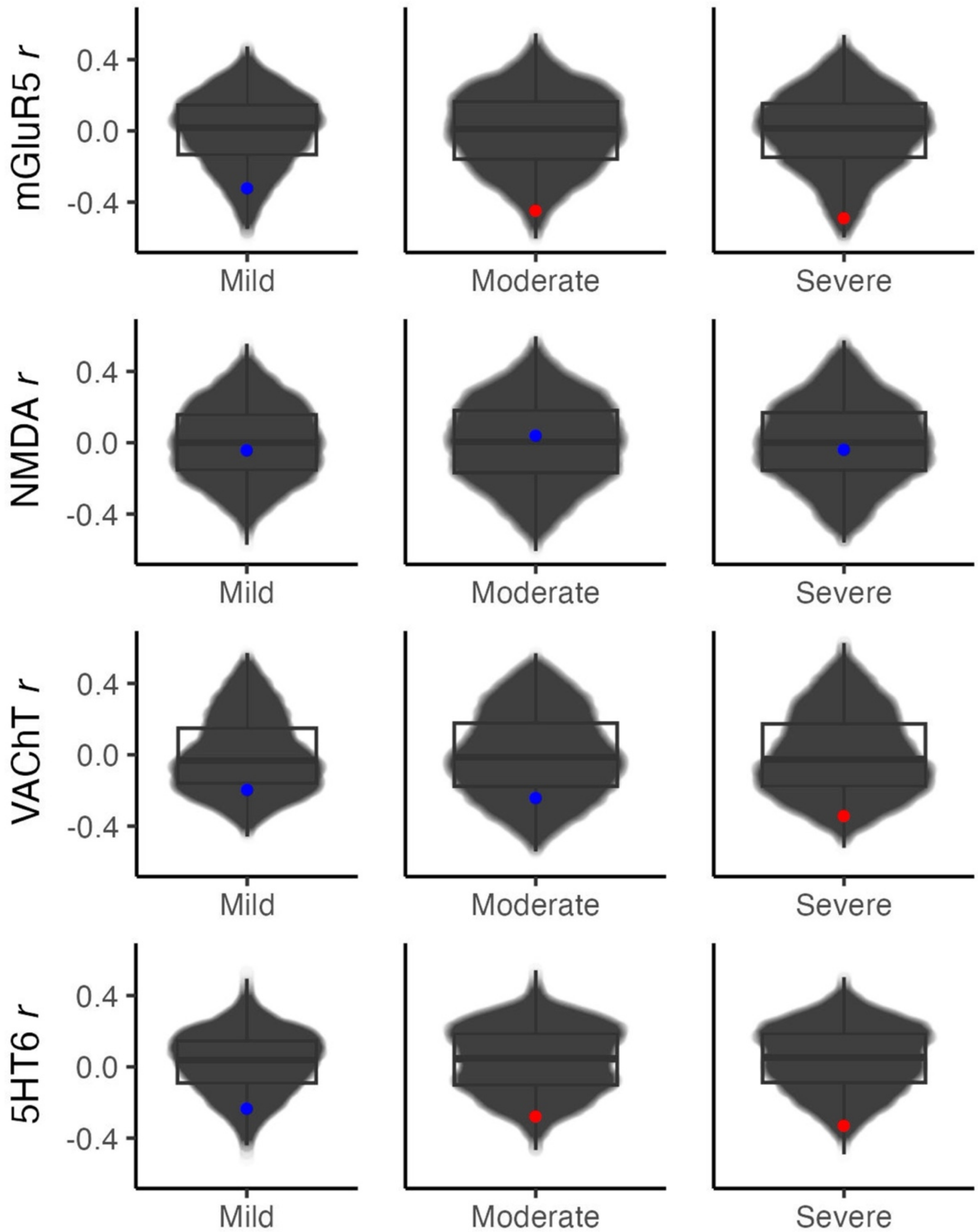

Figure S5. Spin-based permutation tests of the spatial covariance between structural degeneration and receptor maps by disease stage. Black dots represent the spin-based null distribution. Colored dots indicate empirical Pearson's *r* values with red reflecting p_{spin} <= .05 and blue reflecting non-significant p_{spin}. Mild = CDR 0.5, Moderate = CDR 1, Severe = CDR 2 or 3.

Table S8. Association of clinical features and spatial covariance of VAChT density and structural degeneration for each parcellation.

	Predictor	FTLD CDR SOB	DSB Correct	Category fluency	Letter fluency	Figure copy	NPI Severity
Schaefer 100	VAChT	-6.1** (-10,-2.3)	0.77 (-2.6,4.1)	9.9** (3.4,16)	11** (4.0,18)	-0.93 (-5.6,3.7)	-7.9* (-15,-0.59)
	Sex	-0.87 (-2.1,0.34)	0.92 (-0.13,2.0)	1.5 (-0.49,3.6)	1.8 (-0.45,4.0)	2.2** (0.72,3.6)	0.52 (-1.8,2.8)
	Education		0.16 (-0.04,0.36)	0.20 (-0.19,0.58)	0.46* (0.03,0.88)	0.00 (-0.28,0.28)	-0.12 (-0.55,0.31)
	(Intercept)	8.3*** (7.2,9.3)	1.6 (-1.5,4.7)	6.1* (0.06,12)	1.6 (-5.0,8.1)	13*** (8.4,17)	10** (3.7,17)
	R^2	0.102	0.061	0.105	0.136	0.083	0.045
Schaefer 200	VAChT	-7.5** (-12,-3.1)	1.0 (-2.8,4.8)	11** (3.4,18)	11* (2.4,19)	-1.8 (-7.1,3.5)	-8.7* (-17,-0.42)
	Sex	-0.87 (-2.1,0.33)	0.92 (-0.13,2.0)	1.5 (-0.50,3.6)	1.8 (-0.48,4.0)	2.2** (0.72,3.6)	0.52 (-1.8,2.8)
	Education		0.16 (-0.04,0.36)	0.19 (-0.20,0.57)	0.43* (0.01,0.86)	-0.01 (-0.28,0.27)	-0.11 (-0.54,0.32)
	(Intercept)	8.3*** (7.3,9.4)	1.6 (-1.5,4.7)	6.1* (0.03,12)	1.6 (-5.1,8.2)	13*** (8.4,17)	10** (3.7,17)
	R^2	0.114	0.062	0.099	0.111	0.085	0.043
Schaefer 400	VAChT	-8.0** (-13,-3.2)	0.88 (-3.3,5.1)	11** (3.1,20)	11* (2.3,20)	-1.5 (-7.3,4.3)	-9.6* (-19,-0.48)
	Sex	-0.96 (-2.2,0.25)	0.93 (-0.12,2.0)	1.7 (-0.39,3.7)	1.9 (-0.37,4.1)	2.1** (0.70,3.6)	0.41 (-1.9,2.7)
	Education		0.16 (-0.04,0.36)	0.19 (-0.20,0.58)	0.44* (0.01,0.86)	0.00 (-0.28,0.27)	-0.12 (-0.55,0.32)
	(Intercept)	8.3*** (7.3,9.4)	1.6 (-1.5,4.7)	6.0 (-0.05,12)	1.5 (-5.2,8.2)	13*** (8.4,17)	10** (3.7,17)
	R^2	0.109	0.061	0.091	0.108	0.084	0.043
Lausanne	VAChT	-7.8** (-12,-3.2)	1.6 (-2.3,5.6)	11** (3.1,19)	13** (4.1,21)	-0.61 (-6.2,4.9)	-6.5 (-15,2.3)
	Sex	-0.78 (-2.0,0.41)	0.90 (-0.14,1.9)	1.4 (-0.61,3.5)	1.6 (-0.58,3.9)	2.2** (0.72,3.6)	0.59 (-1.7,2.9)
	Education		0.17 (-0.03,0.36)	0.17 (-0.21,0.56)	0.43* (0.01,0.85)	0.00 (-0.27,0.28)	-0.09 (-0.52,0.35)
	(Intercept)	8.4*** (7.4,9.4)	1.6 (-1.5,4.7)	6.2* (0.09,12)	1.6 (-5.0,8.2)	13*** (8.4,17)	10** (3.5,17)
	R^2	0.113	0.065	0.094	0.127	0.082	0.023

β (95% confidence interval) *p<0.05; **p<0.01; ***p<0.001; Abbreviation: SOB = Sum of boxes; DSB = Digit span backwards; NPI = Neuropsychiatric Inventory

Table S9. Association of clinical features and spatial covariance of 5HT6 density and structural degeneration for each parcellation.

	Predictor	FTLD CDR SOB	DSB Correct	Category fluency	Letter fluency	Figure copy	NPI Severity
Schaefer 100	5HT6	-7.6*** (-12,-3.3)	2.4 (-1.3,6.2)	16*** (8.8,23)	16*** (8.5,24)	-0.87 (-6.2,4.4)	4.5 (-3.9,13)
	Sex	-0.71 (-1.9,0.49)	0.86 (-0.18,1.9)	1.2 (-0.77,3.1)	1.4 (-0.76,3.5)	2.2** (0.73,3.6)	0.45 (-1.9,2.8)
	Education		0.18 (-0.02,0.38)	0.27 (-0.10,0.64)	0.52* (0.11,0.93)	0.00 (-0.28,0.28)	-0.01 (-0.45,0.44)
	(Intercept)	7.9*** (6.8,9.0)	1.6 (-1.5,4.7)	6.2* (0.43,12)	1.7 (-4.7,8.0)	13*** (8.4,17)	10** (3.4,17)
	R^2	0.121	0.074	0.184	0.190	0.082	0.013
Schaefer 200	5HT6	-9.9*** (-15,-4.7)	8.0*** (3.7,12)	23*** (15,31)	24*** (15,33)	3.3 (-3.1,9.6)	3.8 (-6.5,14)
	Sex	-0.64 (-1.8,0.54)	0.74 (-0.25,1.7)	1.0 (-0.86,2.9)	1.2 (-0.86,3.2)	2.1** (0.65,3.5)	0.46 (-1.9,2.8)
	Education		0.21* (0.02,0.40)	0.27 (-0.08,0.62)	0.53** (0.14,0.91)	0.03 (-0.25,0.31)	-0.02 (-0.47,0.42)
	(Intercept)	7.7*** (6.6,8.9)	1.8 (-1.1,4.7)	6.8* (1.3,12)	2.3 (-3.7,8.4)	13*** (8.5,17)	10** (3.5,17)
	R^2	0.135	0.169	0.260	0.262	0.091	0.008
Schaefer 400	5HT6	-9.9** (-16,-3.8)	8.8*** (3.7,14)	24*** (14,34)	25*** (15,36)	3.1 (-4.3,11)	6.0 (-5.9,18)
	Sex	-0.73 (-1.9,0.48)	0.80 (-0.19,1.8)	1.2 (-0.71,3.1)	1.4 (-0.69,3.5)	2.1** (0.68,3.6)	0.47 (-1.8,2.8)
	Education		0.20* (0.02,0.39)	0.24 (-0.12,0.61)	0.50* (0.10,0.90)	0.02 (-0.25,0.30)	-0.02 (-0.46,0.42)
	(Intercept)	8.1*** (6.9,9.2)	1.7 (-1.3,4.6)	6.5* (0.86,12)	2.0 (-4.2,8.3)	13*** (8.4,17)	10** (3.5,17)
	R^2	0.105	0.156	0.207	0.223	0.088	0.012
Lausanne	5HT6	-13*** (-20,-6.9)	8.0** (2.5,14)	20*** (9.3,31)	23*** (12,35)	6.8 (-1.0,15)	5.1 (-7.6,18)
	Sex	-0.73 (-1.9,0.44)	0.84 (-0.18,1.8)	1.3 (-0.68,3.3)	1.5 (-0.66,3.7)	2.1** (0.67,3.5)	0.49 (-1.8,2.8)
	Education		0.19* (0.00,0.38)	0.20 (-0.17,0.58)	0.47* (0.06,0.88)	0.04 (-0.23,0.31)	-0.03 (-0.47,0.41)
	(Intercept)	8.2*** (7.1,9.2)	1.5 (-1.5,4.5)	6.1* (0.22,12)	1.6 (-4.8,8.0)	13*** (8.4,17)	10** (3.4,17)
	R^2	0.154	0.130	0.139	0.179	0.108	0.008

β (95% confidence interval) *p<0.05; **p<0.01; ***p<0.001; Abbreviation: SOB = Sum of boxes; DSB = Digit span backwards; NPI = Neuropsychiatric Inventory
